# Widespread distribution of BpfA-mediated bisphenol F degradation among members of the *Pseudomonadota* and *Actinomycetota*

**DOI:** 10.1093/ismejo/wraf206

**Published:** 2025-09-15

**Authors:** Mingliang Zhang, Changchang Wang, Yanni Huang, Qian Li, Junqiang Hu, Kaihua Pan, Qian Zhu, Wankui Jiang, Jiguo Qiu, Xin Yan, Qing Hong

**Affiliations:** Department of Microbiology, College of Life Sciences, Nanjing Agricultural University, Key Laboratory of Agricultural and Environmental Microbiology, Ministry of Agriculture and Rural Affairs, Nanjing 210095, China; Department of Microbiology, College of Life Sciences, Nanjing Agricultural University, Key Laboratory of Agricultural and Environmental Microbiology, Ministry of Agriculture and Rural Affairs, Nanjing 210095, China; Department of Microbiology, College of Life Sciences, Nanjing Agricultural University, Key Laboratory of Agricultural and Environmental Microbiology, Ministry of Agriculture and Rural Affairs, Nanjing 210095, China; Department of Microbiology, College of Life Sciences, Nanjing Agricultural University, Key Laboratory of Agricultural and Environmental Microbiology, Ministry of Agriculture and Rural Affairs, Nanjing 210095, China; Department of Microbiology, College of Life Sciences, Nanjing Agricultural University, Key Laboratory of Agricultural and Environmental Microbiology, Ministry of Agriculture and Rural Affairs, Nanjing 210095, China; Department of Microbiology, College of Life Sciences, Nanjing Agricultural University, Key Laboratory of Agricultural and Environmental Microbiology, Ministry of Agriculture and Rural Affairs, Nanjing 210095, China; Department of Microbiology, College of Life Sciences, Nanjing Agricultural University, Key Laboratory of Agricultural and Environmental Microbiology, Ministry of Agriculture and Rural Affairs, Nanjing 210095, China; State Key Laboratory of Materials-Oriented Chemical Engineering, College of Biotechnology and Pharmaceutical Engineering, Nanjing Tech University, Nanjing 211800, PR China; Department of Microbiology, College of Life Sciences, Nanjing Agricultural University, Key Laboratory of Agricultural and Environmental Microbiology, Ministry of Agriculture and Rural Affairs, Nanjing 210095, China; Department of Microbiology, College of Life Sciences, Nanjing Agricultural University, Key Laboratory of Agricultural and Environmental Microbiology, Ministry of Agriculture and Rural Affairs, Nanjing 210095, China; Department of Microbiology, College of Life Sciences, Nanjing Agricultural University, Key Laboratory of Agricultural and Environmental Microbiology, Ministry of Agriculture and Rural Affairs, Nanjing 210095, China

**Keywords:** bisphenol F, microbial biodegradation, genetic determinant, flavoprotein oxidase BpfA, distribution pattern

## Abstract

Bisphenol F, a widely used primary raw material in the production of polycarbonate and epoxy resins, is frequently detected in the environment and poses significant risks to ecosystems and human health. Microorganisms play an important role in bisphenol F degradation in the natural environment; however, the genetic determinants involved remain unknown. A flavoprotein oxidase BpfA from *Microbacterium* sp. strain F2 was identified in this study, which is responsible for the crucial steps of bisphenol F degradation involving its conversion to 4,4′-dihydroxybenzophenone through three consecutive reactions. BpfA phylogenetically clusters within the 4-phenol oxidizing subfamily of the vanillyl alcohol oxidase/*para*-cresol methylhydroxylase flavoprotein family. Three homologs in this subfamily—vanillyl alcohol oxidase VAO, eugenol oxidase EUGO, and flavoprotein oxidase FBO—shared over 35.0% identity with BpfA and demonstrated bisphenol F-degrading activity, yet the catalytic efficiency of BpfA against bisphenol F (508.1 mM^−1^ s^−1^) was significantly higher than that of vanillyl alcohol oxidase VAO (0.2 mM^−1^ s^−1^), eugenol oxidase EUGO (0.2 mM^−1^ s^−1^), and flavoprotein oxidase FBO (0.3 mM^−1^ s^−1^). Structural analysis indicated that strong active site hydrophobicity was likely the reason for this high catalytic efficiency. Bioinformatics-based taxonomic profiling revealed that candidate bisphenol F degraders carrying *bpfA* mainly belonged to the *Pseudomonadota* and *Actinomycetota* phyla, and were predominantly found in metagenomes from cultivated land and forests. This study elucidated the function and distribution pattern of *bpfA*, enhancing our understanding of microbial bisphenol F degradation in the environment.

## Introduction

Bisphenols (BPs) are monomers used in the resin and plastic industry to produce lacquers and the inner coating of food cans and thermal paper; they include bisphenol A (BPA), bisphenol B (BPB), bisphenol C (BPC), bisphenol E (BPE), bisphenol F (BPF), bisphenol S (BPS), and bisphenol AF (BPAF) (Table S1). Toxicological studies have shown that BPA has endocrine-disrupting effects and may trigger a variety of health problems, such as cancer [1], reproductive disorders [2, 3], and obesity [4, 5]. As one of the main substitutes for BPA, the usage of BPF has increased yearly, becoming a new, widely present pollutant in the environment [6]. BPF enters aquatic, soil, and atmospheric environments through industrial effluent discharge, plastic degradation, and landfill leachate [7, 8]. BPF contamination in East Asian aquatic systems has reached high levels, exceeding 1000 ng L^−1^ in rivers and seawater across Japan, Korea, and China; the highest concentration (2850 ng L^−1^) was detected in Tokyo's Tamagawa River [9–11]. Additionally, the breast milk samples collected from 181 healthy females across various provinces and cities in China contained an average BPF concentration of 0.107 μg L^−1^ [12], suggesting that it may also pose risks to human health through bioaccumulation.

BPF has toxic effects on organisms. Embryonic exposure to 0.5 mM BPF in fruit flies reduced the number of neuroblasts and intermediate neural progenitor cells, leading to an imbalance in the neuron-to-glial cell ratio; this disrupted the proliferation of neural stem cells, resulting in neurodevelopmental toxicity and abnormal larval behaviour [13]. In transcriptomic and metabolomic studies, exposure to 1.0 μM BPF upregulated the expression of pro-inflammatory cytokines (such as interleukin-17A and tumour necrosis factor-α) in human colonic epithelial cells (NCM460), while downregulating tight junction proteins (ZO-1 and CLDN1), disrupting intestinal barrier function [14]. In addition, adult male zebrafish exposed to 500 and 2500 nM BPF exhibited significant reductions in sperm count, decreased levels of serum testosterone, and increased levels of vitellogenin in the liver, indicating that endocrine-disrupting effects were present [15].

Several bacterial strains capable of growing with BPF have recently been identified, including *Pseudomonas* sp. HS-2 [16], *Pseudomonas* sp. ZH-FAD [17], *Sphingobium yanoikuyae* FM-2 [18], *S. yanoikuyae* TYF-1 [19], *Arthrobacter* sp. YC-RL1 [20], and *Microbacterium* sp. F2 [21]. The identification of similar intermediates to those in BPF degradation among strains from various genera has led to the proposal of a common catabolic pathway (Fig. S1). This pathway involved the initial hydroxylation of the BPF carbon bridge to form (4-hydroxyphenyl)methanol (BHPM), followed by dehydrogenation to produce 4,4′-dihydroxybenzophenone (DHBP); subsequently, the Baeyer-Villiger reaction occurred between the two phenolic rings to generate 4-hydroxybenzoic acid-4-hydroxyphenyl ester (HPHB), which was hydrolyzed into *p*-hydroxybenzoic acid (PHBA) and hydroquinone (HQ). These compounds were further degraded to provide carbon sources for microbial growth. Although several BPF-degrading bacterial strains have been reported, the specific genes involved in the conserved metabolic pathway remain unknown. Elucidating these degradation genes is crucial for understanding the transformation and ultimate fate of BPF in the environment. Additionally, understanding the genetic basis of pollutant degradation at the genetic level is essential to develop effective bioremediation strategies, enhance microbial adaptation in contaminated environments, and improve ecosystem resilience.

In this study, a flavoprotein oxidase BpfA was identified in the previously isolated BPF-degrading strain *Microbacterium* sp. F2 [21], which catalyzed the conversion of BPF to DHBP through three consecutive reactions. BpfA was compared with its homologs through biochemical and structural analysis, indicating that many homologs have weak activity toward BPF. These homologs were frequently found among members of the *Pseudomonadota* and *Actinomycetota*, predominantly identified in metagenomes from cultivated land and forests. This study reveals the crucial genetic determinants of BPF degradation, enhancing our understanding of its microbial degradation in the environment.

## Materials and methods

### Chemicals and media

BPA, BPB, BPC, BPE, BPF, BPS, BPAF, DHBP, vanillyl alcohol (VA), 4-n-propylguaiacol (4PG), 4-(methoxymethyl)phenol (4MOP), reduced nicotinamide adenine dinucleotide phosphate (NADPH), reduced nicotinamide adenine dinucleotide (NADH), and flavin adenine dinucleotide (FAD) were purchased from Shanghai Macklin Biochemical Technology Co., Ltd (Shanghai, China). BPF degradation experiments were performed in mineral salts medium (MSM, pH 7.0) [21]. The bacterial strains used in this study were cultured in lysogeny broth (LB, pH 7.0) [21].

### Strains, plasmids, and culture conditions

Details of the bacterial strains and plasmids utilized in this study were provided in Table S2. *Microbacterium* sp. F2 was cultured in LB broth or MSM at 30°C and 180 rpm, unless otherwise specified. *Escherichia coli* strains were cultured in LB broth at 37°C and 200 rpm.

### BPF degradation assay

BPF-induced cells were prepared by harvesting strain F2 cells cultured in LB broth, washing them three times with sterile MSM, and inoculating them into MSM containing 0.10 mM BPF. Cells were collected when half of the BPF was degraded. For BPF-induced cell extracts, cells were washed twice with 20 mM Tris–HCl (pH 8.0), resuspended in 15 ml of the same buffer, and lysed using an ultrasonic processor (UH-650B, Auto Science) at 60.0% intensity in an ice bath, with sonication for 2 s and intervals of 3 s. The lysates were then centrifuged at 12000 × *g* for 20 min at 4°C.

For the BPF degradation experiments, a 3 ml reaction mixture containing 0.10 mM BPF and 1 ml of lysate was prepared in 20 mM Tris–HCl (pH 8.0) at 30°C. The reaction was terminated immediately after completion by boiling for 5 min. Samples were collected every 10 min and analyzed by high-performance liquid chromatography (HPLC) to measure the concentration of BPF.

### Sequencing and analysis

Genomic DNA from strain F2 was extracted using a commercial kit (TIANGEN BIOTECH Co., LTD, Beijing, China). The genome of strain F2 was sequenced by Majorbio Biomedical Technology Co., Ltd (Shanghai, China) using the MiSeq System (Illumina). The raw paired-end reads were trimmed and quality controlled using Trimmomatic with the parameters (SLIDINGWINDOW:4:15 MINLEN:75) (version 0.36) [22]. Clean data obtained from these quality control processes were used for further analysis. Genome assembly was performed using ABySS 2.2.0 with multiple k-mer parameters and obtained optimal assembly results [23]. Subsequently, GapCloser was applied to fill remaining local inner gaps and correct single-base polymorphisms in the final assembly [24]. Gene prediction and annotation were carried out using Glimmer 3.02 and the Rapid Annotation Subsystem Technology (RAST) database [25, 26].

### Purification of BpfA from strain F2 cell extract

Strain F2 was pre-cultured in 100 ml LB broth for 24 h; this starter culture was then used to inoculate five individual 2 L flasks (each containing 1 L LB broth) at 1.0% (v/v), followed by incubation at 30°C at 180 rpm for 48 h. Cultures were harvested by centrifugation at 4°C and 12 000 × *g* for 30 min. The harvested cells were washed three times with 20 mM Tris–HCl (pH 8.0) and lysed using an ultrasonic processor (UH-650B, Auto Science) at 80.0% intensity in an ice bath, with sonication for 3 s and intervals of 5 s. The lysates were then centrifuged at 12000 × *g* for 30 min at 4°C. Purification was conducted at 0–4°C using 20 mM Tris–HCl (pH 8.0) as previously reported [27]. The protein band visualized on sodium dodecyl sulfate-polyacrylamide gel electrophoresis (SDS-PAGE) (Fig. S2, lane 6) was excised and subjected to peptide mass spectrometry analysis using an Ultra-HPLC system (Ultimate 3000 nano, Thermo Scientific) coupled with high-resolution mass spectrometry (Q Exactive plus, Thermo Scientific). The excised protein bands were digested with 0.02 μg·μl^−1^ trypsin in 25 mM NH_4_HCO_3_ buffer, followed by drying and desalting.

The digested peptides were loaded onto a Trap column (100 μm × 20 mm, RP-C_18_, Agilent) with a mobile phase consisting of acetonitrile-water-formic acid (90:9:1, vol/vol/vol) at a flow rate of 3 μL·min^−1^. Peptide fragments were identified using a Q Exactive Plus high-resolution mass spectrometry system with high-energy collision dissociation (normalized collision energy 28) and dynamic exclusion (25 s). The full scan range was set from 350 to 2000 *m/z*. The resulting peptide fragments were matched against the annotated genes from strain F2 to pinpoint sequences with high homology. Subsequently, the resulting protein sequences underwent a BLASTp search against the NCBI non-redundant protein (NR) database. Based on these analyses, the gene encoding flavoprotein oxidase, *bpfA*, was identified as a potential candidate for catalyzing BPF and was selected for further protein expression and in vitro BPF degradation assays.

### Bioinformatic analysis

A BLASTp search was conducted using the BpfA sequence (PV658135) from *Microbacterium* sp. F2 as the query sequence, targeting the NCBI NR database to uncover BpfA homologues. The homologous enzymes obtained from sequence alignment were screened to remove artificial mutants and redundant sequences. A sequence similarity network (SSN) was created utilizing the Enzyme Function Initiative Enzyme Similarity Tool [28]. An all-by-all BLAST was performed on the sequences, and the SSN was subsequently built based on an alignment score cutoff of 90. Visualization of the SSN was accomplished with Cytoscape [29]. For BpfA phylogenetic analysis, homologous sequences were retrieved from the NCBI NR database and aligned using ClustalX [30]. The resultant alignment was transferred to MEGA (version 11.0) [31] for further processing. The phylogenetic tree was subsequently generated employing the neighbor-joining algorithm.

Species-level genome bins (SGBs) were previously constructed from 3304 soil metagenomes, ensuring medium to high quality standards (with completeness exceeding 50.0% and contamination <10.0%); further details are available at the Global Soil MAGs (SMAG) Project website [32]. These SGBs were classified using GTDB-Tk (version 1.6.0), and BpfA was identified through Prodigal, with stringent criteria (identity >35.0%, subject coverage >95.0%, and e-value <1 × 10^−5^) [33]. To explore the global distribution of *bpfA*, all amino acid sequences from SGB and GTDB were compared with BpfA sequence (PV658135) using Diamond (v2.1.6) to obtain a putative BpfA consensus sequence. By integrating classification data from SGBs and GTDB, a comprehensive analysis of BpfA species distribution was accomplished [34]. Additionally, target genomes were classified as either isolated or uncultured (including metagenomic assembly genomes and single amplified genomes) by searching both GTDB and NCBI RefSeq [35, 36].

### Gene expression and recombinant enzyme purification

The primer pairs *bpfA*_F2_-F and *bpfA*_F2_-R (Table S3) were used to amplify *bpfA* from strain F2. The amplified fragment was cloned into an NdeI/XhoI-digested pET29a(+) plasmid using homologous recombination, resulting in recombinant plasmid pET-*bpfA*. This plasmid was then introduced into *E. coli* BL21(DE3) to create the expression strain *E. coli* BL21(pET-*bpfA*). *E. coli* BL21(pET-*bpfA*) was inoculated into LB containing 50 mg·L^−1^ kanamycin and cultured until the cell density reached an optical density at 600 nm (OD_600_) of 0.5–0.6, at which point 0.2 mM isopropyl *β*-D-1-thiogalactopyranoside was added to induce protein expression for 12 h at 16°C. Cells were centrifuged and lysed using an ultrasonic processor. The supernatant was obtained by centrifugation at 4°C and then passed through a Ni-NTA column (Sangon, Shanghai, China). A gradient of concentrations of imidazole was used to elute and purify the recombinant protein, which was then dialyzed overnight in Tris–HCl (pH 8.0) and analyzed by SDS-PAGE. A bicinchoninic acid (BCA) kit from Vazyme Biotech Co., Ltd (Nanjing, China) was used to determine the protein concentration.

The eugenol oxidase gene *eugO* from *Rhodococcus jostii* RHA1 (Q0SBK1), VA oxidase gene *vaO* from *Penicillium simplicissimum* CBS 170.90 (P56216), and flavoprotein oxidase gene *fbO* from *Sphingobium* sp. W15 (PV658134) were synthesized by Nanjing Qingke Biotechnology Co., Ltd (Nanjing, China). The protein expression and purification methods for EUGO, VAO, and FBO were similar to those described above.

### Enzyme activity assays

BpfA activity assays were performed at 30°C for 2 h in a 1 ml reaction mixture containing 20 mM Tris–HCl (pH 8.0), 0.10 mM BPF, and 3.0 μM BpfA. The enzymatic reactions were terminated by boiling for 5 min. Enzyme activity was quantified as the amount of enzyme needed to convert 1 μmol of BPF per minute, defined as one unit.

To determine the optimal reaction temperature, BpfA activity was tested at various temperatures, spanning from 20°C to 50°C in 5°C increments. The thermal stability of BpfA was evaluated by subjecting it to different temperatures for 30 min, after which residual activity was assessed as previously described. Non-heated enzyme was used as the control. The optimal reaction pH was determined using a variety of buffers: 20 mM citrate–phosphate (pH 4.0–6.0), 20 mM Tris–HCl (pH 6.0–8.0), and 20 mM glycine-NaOH buffer (pH 8.0–10.0). The pH stability of BpfA was examined by incubating the enzyme at 4°C for 30 min in buffers with diverse pH, subsequently measuring residual activities. Samples were collected prior to complete substrate consumption. A reaction mixture lacking the enzyme served as a negative control. The activity of the reference enzyme was designated as 100.0%, and the relative activities of each reaction were determined as a percentage relative to the reference. Enzyme kinetics for BpfA, EUGO, VAO, and FBO were studied using different concentrations of substrates (BPF, VA, 4PG, and 4MOP). The *K*_m_ and *k*_cat_ values were calculated using the Michaelis–Menten equation with Origin 2018 [37].

To evaluate the substrate specificity of BpfA, it was tested with various substrates (BPA, BPB, BPC, BPE, BPS, BPAF, VA, 4PG, and 4MOP; 0.10 mM each) under the reaction conditions described above. The activity assays for EUGO, VAO, and FBO against BPF were similarly performed following the same procedures, with the exception that FBO assays required 0.2 mM FAD supplementation. Statistical analyses were performed using one-way analysis of variance, with mean comparisons conducted using the least significant difference (LSD) test. All analyses are performed using the Statistical Package for the Social Sciences (SPSS, version 20.0).

### Site-directed mutagenesis

Mutations at specific sites within *bpfA* were engineered using overlap extension PCR. The primer pairs Y93A-F/R, Y474A-F/R, R475A-F/R, D152A-F/R, and H393A-F/R were used to introduce mutation sites (Table S3). The site-directed mutagenesis amplification was carried out following the protocol provided with the site-directed mutagenesis kit (Vazyme Biotech Co., Ltd., China). The resulting amplicons were purified by gel extraction and ligated into pET-29a(+), following the previously described method [38]. The activity assessment of the mutant proteins was carried out using the previously detailed procedures.

### Molecular docking

The structural models of BpfA and FBO were built in AlphaFold3 (https://golgi.sandbox.google.com/) [39], and the 3D structure of BPF is found in PubChem (https://pubchem.ncbi.nlm.nih.gov/). The structural optimization of the BpfA and FBO structural models was carried out using the Amber14 force field [40]. Optimization was conducted in two stages: initially, 2000 steps of steepest descent minimization were performed, followed by 2000 steps of conjugate gradient minimization to further refine the structure. The final optimized structures were used as the models for subsequent analysis.

VAO was modeled using the crystal structure 1VAO [41], while EUGO was modeled using the crystal structure 5FXD [42]. Molecular docking was performed using AutoDock4.2.6 [43] to obtain the structure of the BPF, which was then hydrogenated and optimized using MOPAC with PM3 atomic charges calculated [44]. Finally, the structures of the ligand and receptor were processed using Autodock Tools 1.5.6 [45]. The patterns of the binding of BPF to BpfA, VAO, EUGO, and FBO were visualized by PyMOL 3.1 [46]. Visualization of the interactions between the protein and the substrate was performed using LigPlot+ [47].

### Analytical methods

Enzyme assay samples were boiled for 5 min and subsequently centrifuged at 12000 × *g* for 5 min. The resulting supernatant was passed through a 0.2-μm pore membrane before further analysis of the products. The detection and quantification of BPF and its metabolites were performed using HPLC on a Dionex UltiMate 3000 system (Thermo Fisher Scientific, Waltham, MA, USA) equipped with a C_18_ reverse-phase column (4.6 × 250 mm, 5.0 μm). The mobile phase consisted of methanol–water-acetic acid (60:39:1, vol/vol/vol) with a flow rate of 0.8 mL·min^−1^. Elution from the column was monitored by measuring the absorbance at 270 nm. The injection volume was 20.0 μl, and the column temperature was maintained at 40°C.

The identification of BpfA and its metabolites were performed using mass spectrometry on an AB SCIEX Triple TOF 5600 plus high-resolution mass spectrometry system equipped with a Turbo V probe. All metabolites were electrospray ionized (positive and negative polarity), and the scan range was set from 100 to 800 *m/z*. The electrospray ionization (ESI) voltage was set to +5500 V for positive ion mode and − 4500 V for negative ion mode. The collision energy was set to 45 V. The HPLC detection conditions for 4MOP were identical to those for BPF. For BPA, BPB, BPC, BPE, BPS, and BPAF, the mobile phase was acetonitrile-water (50:50, vol/vol); for 4PG, the mobile phase was acetonitrile-water (70:30, vol/vol), and for VA, the mobile phase was methanol–water-acetic acid (45:54:1, vol/vol/vol). All other HPLC parameters for BPA, BPB, BPC, BPE, BPS, and BPAF, 4PG, and VA were the same as those used for BPF detection.

The content of hydrogen peroxide (H_2_O_2_) generated during the degradation of BPF by BpfA was quantified using the H_2_O_2_ colorimetric method [48], which involved the oxidation of Fe^2+^ to Fe^3+^ under acidic conditions, followed by the formation of a Fe^3+^-dye complex with a maximum absorption at 590 nm, with the absorbance being proportional to the concentration of H_2_O_2_. Enzyme assay samples were incubated with a reaction mixture containing Fe^2+^ at room temperature for 20 min, and the absorbance at 590 nm was measured using a Multiskan GO microplate spectrophotometer (Thermo Fisher Scientific). The H_2_O_2_ concentration was then calculated based on a standard curve.

## Results and discussion

### Cloning and sequence analysis of *bpfA*

*Microbacterium* sp. F2 has been reported to use BPF as the sole carbon source for growth, with degradation being BPF-induced [21]. Cell extract from BPF-induced strain F2 degraded 0.10 mM BPF to below the detection limit (0.5 μM) within 30 min, regardless of whether NADPH, NADH, and FAD were introduced (Fig. S3). The gene responsible for BPF degradation was identified by conducting peptide mass spectrometry on protein fragments from the cell extract of strain F2, followed by matching these fragments to the genomic sequence of strain F2, and ultimately confirmed through functional validation. A four-stage purification protocol was utilized to purify the BPF-degrading enzyme from cell extract of the BPF-induced strain F2. Initially, the specific activity in the cell extract was determined to be 0.37 U·mg^−1^ protein. Following the four purification steps, the specific activity of the final fraction rose to 14.74 U·mg^−1^, with a 39.84-fold increase in specific activity and a recovery rate of 4.8% (Table S4). SDS-PAGE analysis of the final fraction showed a single protein band (Fig. S2), which was excised and subjected to peptide mass spectrometry analysis. The analysis yielded sequences of 15 peptide fragments, which were then matched against all annotated genes within strain F2 (JBNVBV000000000) (Table S5). Subsequently, all candidate genes predicted to degrade BPF were annotated and experimentally validated. A flavoprotein oxidase encoded by *orf1733* (ACQVDU_06965) demonstrated BPF-degrading activity and was designated as BpfA, which was selected for further studies.

**Figure 1:**
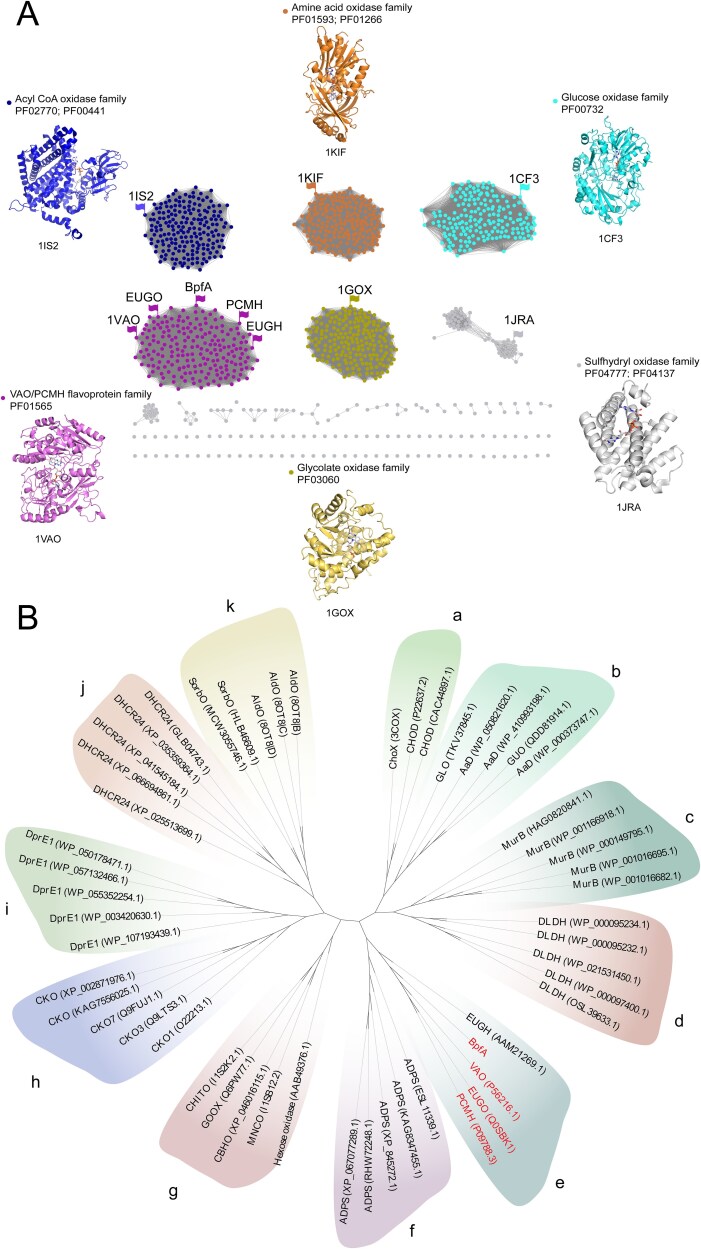
(A) Sequence similarity network (SSN) for representative flavoprotein oxidase superfamily in the NCBI non-redundant protein sequences database. Each item is arranged in the following order: Protein name and accession number. Around them are shown the structures of representative members of the different flavin-dependent oxidases. (B) Phylogenetic analysis of BpfA with sequences of other members of the vanillyl alcohol oxidase/*Para*-cresol methylhydroxylase (VAO/PCMH) flavoprotein family. Subfamilies are marked with distinct letters (A-K) as indicated in the figure and defined below: A: Cholesterol oxidases subfamily; B: Aldonolactone oxidoreductases subfamily; C: MurBs subfamily; D: α-Hydroxy acid dehydrogenases subfamily; E: 4-phenol oxidizing subfamily; F: Alkyl-dihydroxyacetone phosphate synthases subfamily; J: BBE-like subfamily; H: Cytokinin dehydrogenases subfamily; I: DprE1s subfamily; J: Sterol reductases subfamily; K: Alditol oxidases subfamily.

The *bpfA* (1599 bp) encoded a 532-amino acid oxidase that shared 30.0%–55.0% identity with flavoprotein oxidases from various genera (NR database). BpfA was classified within the flavoprotein oxidase superfamily [49, 50]; SSN analysis indicated that BpfA was a member of the VA oxidase/*para*-cresol methylhydroxylase (VAO/PCMH) flavoprotein family (PF01565) within this superfamily (Fig. 1A). The VAO/PCMH flavoprotein family was further divided into 11 subfamilies [51]. BpfA clustered with the reported members of the 4-phenol oxidizing (4PO) subfamily (Fig. 1B), including eugenol oxidase EUGO (Q0SBK1) [52], VAO (P56216) [53], and PCMH (P09788.3) [54].

Enzymes of the 4PO subfamily contain five characteristic motifs (Fig. S4) [51, 55, 56], among which five amino acids are crucial for catalysis. Tyr93, Tyr474, and Arg475 (BpfA numbering) are proposed to be essentially involved in the deprotonation of substrates upon their arrival in the active site [53], with Tyr93 and Tyr474 forming hydrogen bonds with the substrate BPF. His393 contributes to the oxidation capability of FAD by covalently binding to the flavin, increasing its redox potential [57]. Asp152 does not directly participate in the formation of the covalent bond, but stabilizes the conformation of His393 through charge interaction, facilitating the autocatalytic formation of the covalent bond FAD to His393 [58].

### Functional identification of BpfA

**Figure 2:**
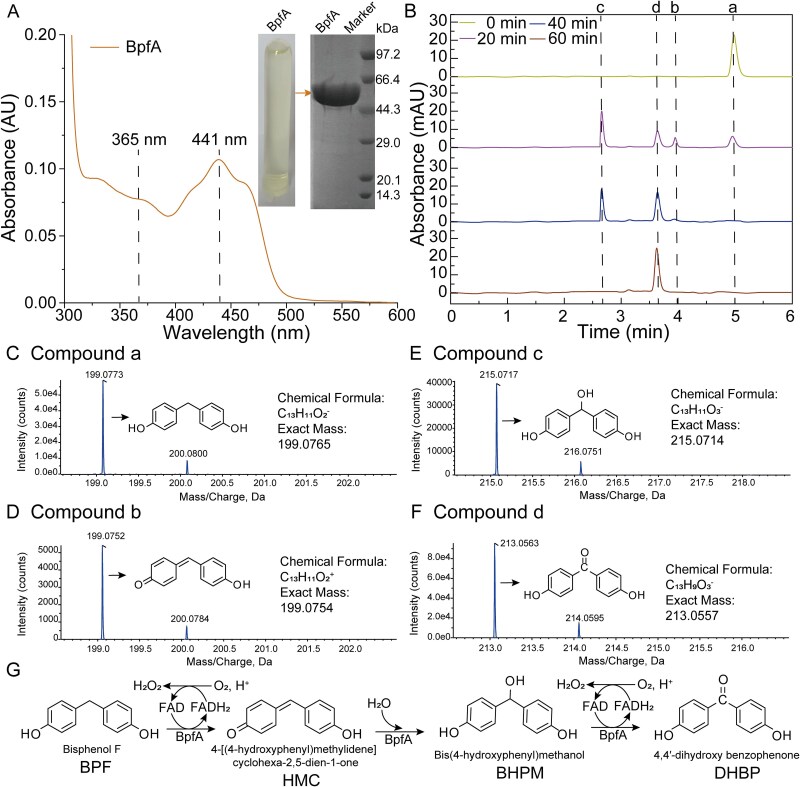
HPLC-MS analysis of BpfA-mediated BPF degradation. (A) UV–vis spectrum of native BpfA (1.40 mM). The inset shows SDS-PAGE analysis of purified BpfA. (B) HPLC analysis of metabolites that appear during the conversion of BPF by purified BpfA. (C) HPLC-MS analysis of compound a (*m/z* 199.0773 [M-H]^−^), which is identified as BPF. (D) HPLC-MS analysis of compound b (*m/z* 199.0752 [M + H]^+^), which is identified as HMC. (E) HPLC-MS analysis of compound c (*m/z* 215.0717 [M-H]^−^), which is identified as BHPM. (F) HPLC-MS analysis of compound d (*m/z* 213.0563 [M-H]^−^), which is identified as DHBP. (G) The proposed enzymatic reaction mechanism of BPF degradation by BpfA. BPF: Bisphenol F; HMC: 4-[(4-hydroxyphenyl)methylidene]cyclohexa-2,5-dien-1-one; BHPM: Bis (4-hydroxyphenyl) methanol; DHBP: 4,4′-dihydroxybenzophenone.

Recombinant BpfA, with an observed molecular mass of 59.9 kDa, was purified and appeared as pale yellow fractions, indicating flavin incorporation. The protein had a typical flavoprotein absorbance spectrum with absorption maxima at 365 and 441 nm (Fig. 2A) [59]. The highest BPF activity was observed at 30°C and pH 8.0 (Fig. S5). When the enzyme was exposed to 40°C for 30 min, it maintained over 80.0% of its initial activity. In contrast, incubation at 45°C for the same duration resulted in significant instability, with the enzyme retaining only 30.0% of its initial activity (Fig. S5A). When stored for 30 min at pH 7.0–10.0, its activity remains >70.0% (Fig. S5B).

Four compounds were detected during BPF degradation, including parental compound BPF (compound a) and three metabolites (compounds b, c, and d) (Fig. 2B). These compounds were identified by HPLC-MS as BPF (compound a, Fig. 2C), 4-[(4-hydroxyphenyl)methylidene]cyclohexa-2,5-dien-1-one (compound b, HMC, Fig. 2D), BHPM (compound c, Fig. 2E), and DHBP (compound d, Fig. 2F). HMC was not detected in the degradation pathway of strain F2 [21], likely because it existed as a transient intermediate during catalysis. This phenomenon was also observed during VAO-mediated degradation of 4MOP [60].

Based on these metabolites and the catalytic mechanisms of 4PO subfamily, a BpfA-mediated catalytic mechanism for BPF was proposed (Fig. 2G). Initially, the phenolic -OH group of BPF was deprotonated by two conserved tyrosine residues of BpfA (Tyr93 and Tyr474). The electron density was then conducted through the aromatic ring, extruding a hydride ion (H^−^) from the α-position, which was accepted by the isoalloxazine ring of the oxidized flavin cofactor (FAD, yellow), generating the reduced flavin cofactor (FADH_2_, colorless). The resulting quinone methide intermediate (HMC) then acted as an electrophile, undergoing nucleophilic addition with a water molecule catalyzed in the BpfA's active site, leading to the formation of the hydroxylated product (BHPM). Finally, BHPM was oxidatively dehydrogenated to yield DHBP. The reduced flavin cofactor (FADH_2_) was reoxidized by molecular oxygen, converting oxygen into H_2_O_2_. According to this mechanism, the conversion of one molecule of BPF by BpfA would produce two molecules of H_2_O_2_. Consistent with this stoichiometry, quantitative analysis using the H_2_O_2_ colorimetric assay [48] detected 0.189 mM H_2_O_2_ production during degradation of 0.10 mM BPF, yielding a molar ratio of approximately 1:2 (BPF: H_2_O_2_) (Fig. S6).

**Figure 3:**
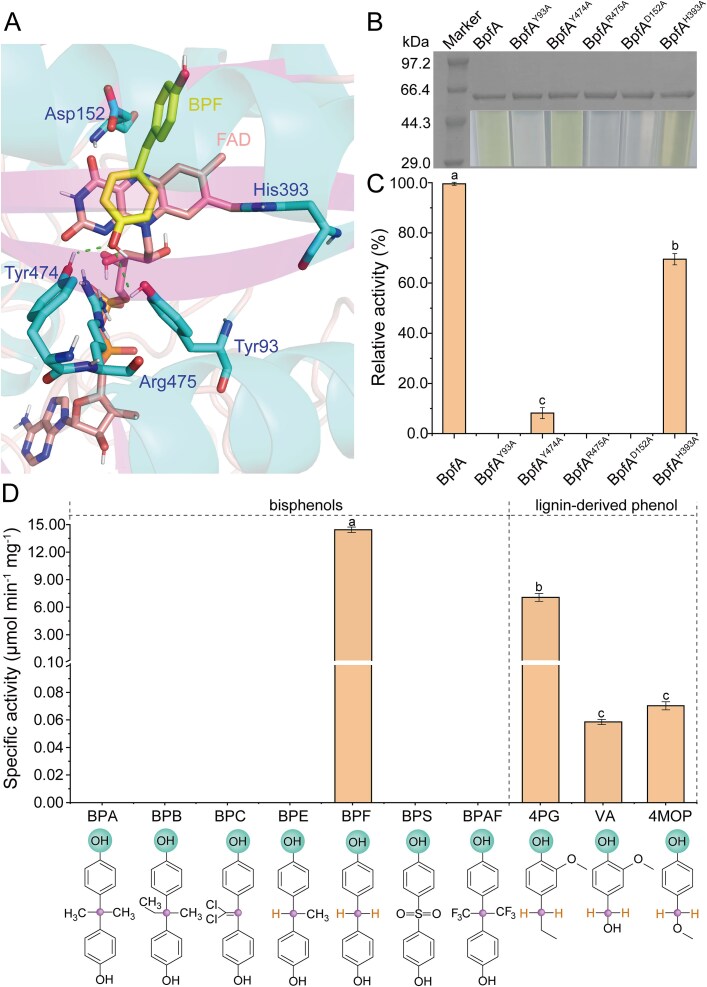
Functional analysis of catalytic residues and substrate specificity of BpfA. (A) Molecular docking of BpfA with BPF. Key catalytic residues are highlighted: the Tyr-Tyr-Arg triad (Y93, Y474, R475) and the conserved FAD-binding residues (D152, H393). Dashed lines represent hydrogen bonds between BpfA and BPF. (B) SDS-PAGE analysis of the purified BpfA and its mutants. Lane 1: Protein marker; lane 2: Wild-type BpfA; lane 3: BpfA^Y93A^; lane 4: BpfA^Y474A^; lane 5: BpfA^R475A^; lane 6: BpfA^D152A^; lane 7: BpfA^H393A^. (C) Assessment of BPF-degrading activity in wild-type BpfA and its variants (BpfA^Y93A^, BpfA^Y474A^, BpfA^R475A^, BpfA^D152A^, and BpfA^H393A^). (D) the specific activity of BpfA against bisphenol compounds (BPA, BPB, BPC, BPE, BPF, and BPAF) and lignin-derived phenolic compounds (4PG, VA, and 4MOP). Substrate structures are presented at the bottom of the figure. The key structural features in the figure are as follows: conserved chemical groups, catalytic sites, and the active hydrogen atoms on the α-carbon of para-substituted phenolic compounds. Data represent the mean values of two replicates using independent enzyme preparation. Values with different lowercase letters are significantly different at *P* < 0.05 according to the LSD test.

As mentioned above, Tyr93, Tyr474, Arg475, Asp152, and His393 played critical roles in the catalytic mechanism of BpfA, facilitating substrate deprotonation and stabilization (Fig. 3A). To validate their roles in BPF degradation, the Tyr-Tyr-Arg triad (Y93, Y474, and R475) and the conserved FAD-binding domain (D152 and H393) of BpfA were replaced by alanine, resulting in five variants (BpfA^Y93A^, BpfA^Y474A^, BpfA^R475A^, BpfA^D152A^, and BpfA^H393A^) (Fig. 3B). Among the five mutants, only purified BpfA^Y474A^ and BpfA^H393A^ exhibited the same pale yellow as the wild-type BpfA (Fig. 3B). BpfA^Y474A^ and BpfA^H393A^ retained partial activity (6.6% and 71.1%, respectively), whereas all other mutants completely lost catalytic function (Fig. 3C). This indirectly highlighted the significance of the Tyr-Tyr-Arg triad for BpfA functionality and also indicated that Asp152 played a more crucial role than His393 in forming a covalent bond with FAD. The results confirmed that BpfA belonged to the 4PO subfamily, featuring the Tyr-Tyr-Arg triad and a conserved FAD-binding domain.

### Substrate spectrum analysis of BpfA

The specific activity of BpfA was 14.72 U·mg^−1^ protein for BPF (Fig. 3D). In addition to BPF, BpfA exhibited no detectable activity against other BPs, such as BPA, BPB, BPC, BPE, BPS, and BPAF (Fig. 3D). BpfA also showed catalytic activity against other *para*-substituted phenolic compounds, which were products of lignocellulosic biomass reductive catalytic fractionation [61], including VA, 4PG, and 4MOP (Fig. 3D). The specific activity of BpfA against BPF was 249.5, 2.1, and 210.3 times higher than that against VA, 4PG, and 4MOP, respectively.

BpfA not only exhibited high degradative activity against BPF but also showed some activity towards 4-alkylphenols, such as VA, 4PG, and 4MOP. It was precisely this enzymatic promiscuity of BpfA that made it a potential biocatalyst for converting 4-alkylphenol compounds into valuable phenolic monomers. In addition, BpfA showed no activity against other BPs (such as BPA, BPB, BPC, BPE, BPS, and BPAF). These results indicated that the presence of two active hydrogen atoms on the α-position carbon of *para*-substituted phenolic compounds was crucial for BpfA to function.

### BpfA and 4PO subfamily proteins

**Figure 4:**
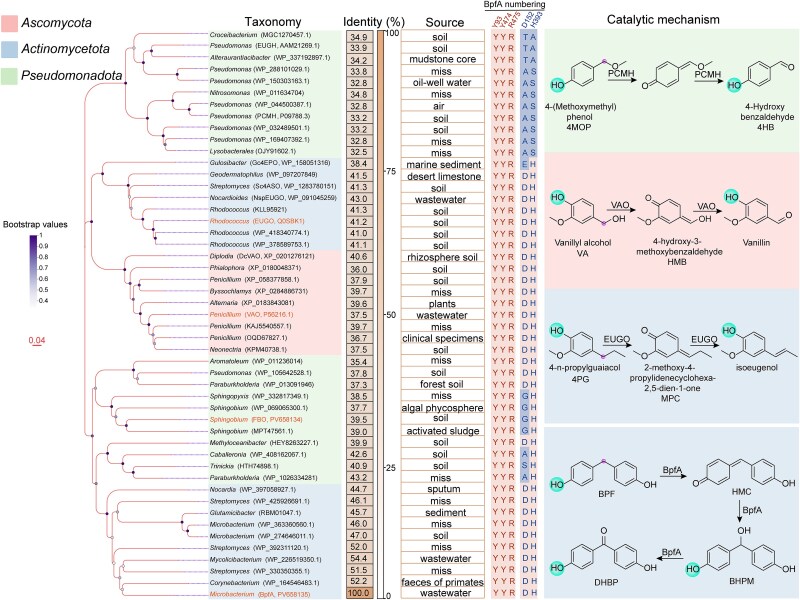
Phylogenetic tree of 4-phenol oxidizing (4PO) subfamily proteins. The identity of BpfA homologous sequences is indicated on the right side of the phylogenetic tree. The three conserved key amino acid residues (Y93, Y474, and R475) and two FAD-binding sites (D152 and H393) in each sequence are shown on the right side; their positions in BpfA are indicated at the top of the columns. The sources of strains containing BpfA homologous sequences are listed to the left of the conserved amino acid sequences. On the far right side of the phylogenetic tree, details of the catalytic mechanisms of VAO, BpfA, EUGO, and PCMH for their respective substrates are shown. Scale bar indicates an evolutionary distance of 0.04.

This study investigated whether homologs of BpfA in the 4PO subfamily could catalyze BPF. The subfamily was divided into oxidases (using O_2_, VAO and EUGO) and dehydrogenases (using cytochrome c, PCMH and eugenol hydroxylase EUGH) based on their electron acceptor preference [41, 42, 54, 62, 63]. Phylogenetic analysis revealed an early divergence between these two major clades (Fig. 4). Within the oxidase clade, BpfA, VAO, EUGO, and flavoprotein oxidase FBO resided on distinct branches (Fig. 4), suggesting independent evolutionary origins. Based on the sequence similarity and coverage between BpfA and the well-studied oxidase representatives VAO (37.5% identity and 96.0% coverage) and EUGO (41.1% identity and 98.0% coverage), a threshold range (sequence identity >35.0% and subject coverage >95.0%) was defined for candidate selection. To validate this threshold, VAO, EUGO, as well as FBO (from a different oxidase branch within this range, with 39.5% identity and 97.0% coverage), were synthesized for subsequent functional characterization. This strategy provides a basis for screening potential *bpfA* gene in metagenomic databases. Additionally, the dehydrogenase PCMH was not synthesized for functional validation, as it employs a different electron acceptor mechanism and has a complex two-component structure [54].

Recombinant VAO and EUGO were purified and appeared pale yellow, exhibiting a typical flavoprotein absorbance spectrum with absorption maxima at 365 and 441 nm (Fig. S7A,B), respectively. In contrast, the purified FBO was colorless and lacked the typical flavoprotein absorbance spectrum (Fig. S7C). Sequence alignment revealed a single amino acid substitution (D to G) within the FAD-binding motif of FBO (Fig. 4), suggesting impaired FAD binding. This defect likely explained its atypical appearance and indicated that FBO might require the addition of FAD to function properly. Subsequent experiments confirmed this: the addition of FAD significantly enhanced the catalytic activity of FBO towards BPF (*P* < 0.05) but had no effect on the activities of BpfA, VAO, or EUGO. Regardless of FAD supplementation, VAO, EUGO, and FBO exhibited relatively weak degradation activity against BPF, while the specific activity of BpfA was significantly higher than that of these enzymes (*P* < 0.05) (Fig. S7D). These results demonstrated that 4PO subfamily members within the defined threshold range (sequence identity >35%, subject coverage >95.0%) showed catalytic activity against BPF, thereby validating the screening strategy for identifying functional homologs.

Potential catalytic differences among BpfA, VAO, EUGO, and FBO toward BPF, 4MOP, VA, and 4PG were investigated by determining the enzymatic kinetic parameters for each enzyme-substrate pair. BpfA, VAO, and EUGO were all capable of catalyzing all four substrates (Table S6). When BPF was used as the substrate, the catalytic efficiency (*k*_cat_/*K*_m_) of BpfA (508.1 mM^−1^·s^−1^) was significantly higher than that of VAO (0.2 mM^−1^·s^−1^), EUGO (0.2 mM^−1^·s^−1^), and FBO (0.3 mM^−1^·s^−1^) (*P* < 0.05), while no significant differences were observed among VAO, EUGO, and FBO. With 4MOP as the substrate, the catalytic efficiencies of VAO (54.2 mM^−1^·s^−1^) and EUGO (56.0 mM^−1^·s^−1^) were significantly higher than that of BpfA (4.0 mM^−1^·s^−1^) (*P* < 0.05), while FBO exhibited no detectable activity. For VA, the catalytic efficiency of EUGO (65.8 mM^−1^·s^−1^) was significantly higher than that of VAO (42.9 mM^−1^·s^−1^), BpfA (3.3 mM^−1^·s^−1^), and FBO (0.1 mM^−1^·s^−1^) (*P* < 0.05). When 4PG was the substrate, the catalytic efficiency of BpfA (224.4 mM^−1^·s^−1^) was significantly higher than that of VAO (70.4 mM^−1^·s^−1^), EUGO (80.9 mM^−1^·s^−1^), and FBO (0.2 mM^−1^·s^−1^) (*P* < 0.05), with no significant difference between VAO and EUGO. BpfA's substrate affinity (*K*_m_) followed the order: BPF (12.4 ± 1.9) μM > 4PG (25.4 ± 2.9) μM > VA (265.9 ± 10.3) μM > 4MOP (297.4 ± 5.0) μM; its catalytic efficiency towards BPF was also highest, indicating that BPF is the optimal substrate for BpfA.

Building on our kinetic analyses, molecular docking was performed to elucidate the structural basis for differential catalytic efficiency toward BPF among BpfA, VAO, EUGO, and FBO. The consistent binding of BPF near the FAD cofactor in all four proteins suggested a similar catalytic mechanism. (Fig. S8). However, the binding energies for BPF with BpfA, FBO, VAO, and EUGO were − 6.928, −6.385, −4.284, and − 3.929 kcal·mol^−1^, respectively, indicating that BPF exhibited the strongest binding affinity for BpfA, followed by FBO, while VAO and EUGO showed comparatively weaker affinities for BPF.

**Figure 5:**
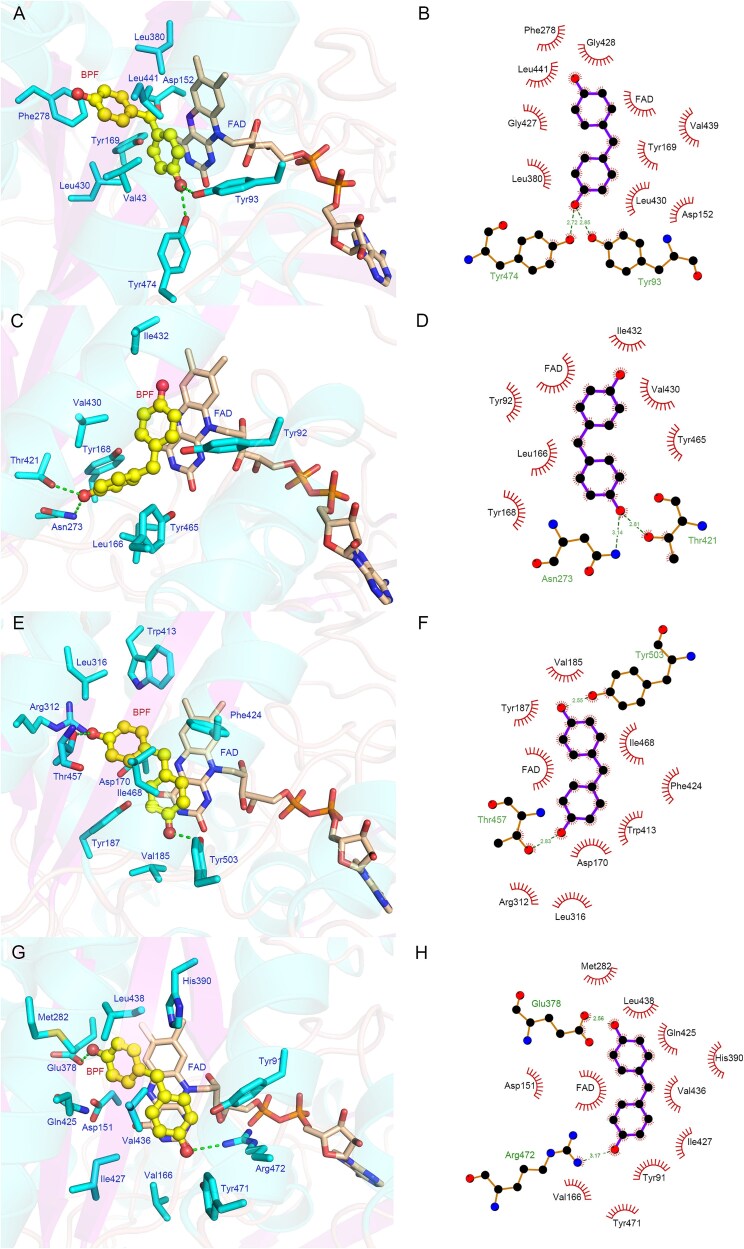
Molecular binding modes of four proteins with BPF. (A) Interaction between BpfA and BPF protein amino acids, with dashed lines indicating hydrogen bonds. (B) Two-dimensional interaction between BpfA and BPF, with dashed lines representing hydrogen bonds and serrated lines indicating hydrophobic interactions. (C) Interaction between FBO and BPF protein amino acids, with dashed lines indicating hydrogen bonds. (D) Two-dimensional interaction between FBO and BPF, with dashed lines representing hydrogen bonds and serrated lines indicating hydrophobic interactions. (E) Interaction between VAO and BPF protein amino acids, with dashed lines indicating hydrogen bonds. (F) Two-dimensional interaction between VAO and BPF, with dashed lines representing hydrogen bonds and serrated lines indicating hydrophobic interactions. (G) Interaction between EUGO and BPF protein amino acids, with dashed lines indicating hydrogen bonds. (H) Two-dimensional interaction between EUGO and BPF, with dashed lines representing hydrogen bonds and serrated lines indicating hydrophobic interactions.

To elucidate the driving forces behind the binding of BPF to the four proteins, their interaction modes were analyzed (Fig. 5). BPF bound near the FAD in the structure of BpfA (Fig. 5A,B), forming hydrogen bonds with Tyr93 and Tyr474. Additionally, the benzene ring of BPF could form strong pi-pi interactions with the aromatic ring of FAD. The surroundings of BPF included numerous aromatic amino acids (Tyr93, Tyr169, Phe278, and Tyr474) and hydrophobic amino acids (Val43, Leu380, and Leu430). Their large side chains might sterically restrict BPF movement, thereby stabilizing its binding near FAD and the catalytic site, and facilitating catalysis. In FBO, Asn273 and Thr421 formed hydrogen bonds with BPF. Steric constraints in FBO's catalytic pocket displaced Tyr92 and Tyr465 > 5 Å from BPF—beyond hydrogen-bonding distance—preventing the catalytic Tyr-Tyr-Arg triad from engaging the substrate. Consequently, FBO depended on non-catalytic hydrogen bonds from Asn273 and Thr421 for substrate anchoring. This contrasted with BpfA, where Tyr93 and Tyr474 directly mediated proton transfer to activate BPF's phenolic group, a functional dichotomy explaining FBO's reduced catalytic turnover (Fig. 5C,D). In VAO, Thr457 and Tyr503 formed hydrogen bonds with BPF. While Tyr503 (structurally equivalent to BpfA's Tyr474) bound BPF, the catalytic triad's proton-abstracting residue (Tyr108) failed to engage, preventing efficient phenolic deprotonation. In addition, the BPF was surrounded by numerous aromatic amino acids (Tyr187, Trp413, Phe424, and Tyr503) and a small amount of hydrophobic amino acids (Val185 and Leu316). Compared with the BpfA and VAO, VAO had relatively fewer hydrophobic amino acids interacting with BPF and more hydrophilic amino acids (Asp170, Arg312, and Thr457) (Fig. 5E,F). These hydrophilic amino acids might alter the nature of the binding site, affecting BPF binding. In EUGO, Glu378 and Arg472 formed hydrogen bonds with BPF. Electrostatic stabilization by Arg472 (corresponding to BpfA's Arg475) preferentially anchored BPF's phenolic oxygen, suppressing deprotonation. Similar to VAO, EUGO also had a higher number of hydrophilic amino acids (Asp151, Glu378, Gln425, and Arg472) interacting with BPF (Fig. 5G, H).

In summary, BPF could bind to the active sites of all four proteins, with the strongest binding affinity for BpfA and relatively weaker affinities for VAO and EUGO. The binding modes of BPF with these four proteins suggested that the weaker affinities of VAO and EUGO might be due to their relatively weaker hydrophobicity and stronger hydrophilicity at the active sites, which was consistent with BPF's strong hydrophobic nature and its tendency to interact more readily with hydrophobic amino acids.

### Global survey of the environmental distributions of *bpfA*

**Figure 6:**
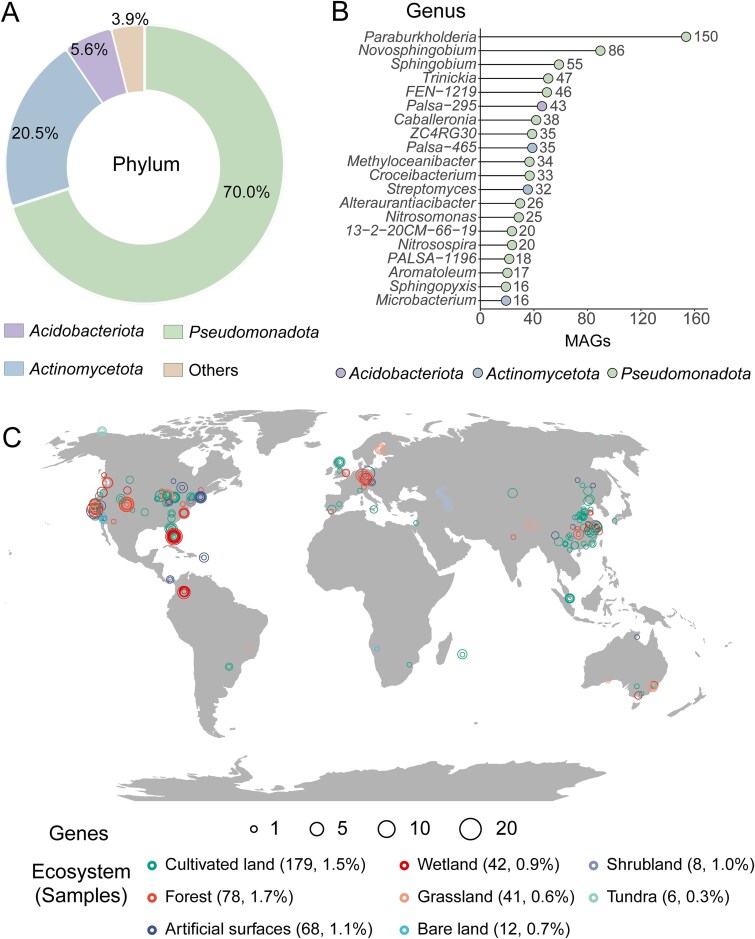
Microbial classification and distribution pattern of *bpfA* in global environments. (A) The number of MAGs maps *bpfA* across phyla (top 3) in the domain of bacteria. (B) The number of MAGs maps *bpfA* across genera (top 20) in the domain of bacteria. (C) Geographic and relative abundance distribution of *bpfA* in each sample. The relative abundance of *bpfA*-containing MAGs across ecosystems is shown in Table S7.

Based on the established sequence threshold (identity >35.0% and coverage >95.0%) for functional screening, the environmental distribution of BpfA homologs was surveyed as a proxy for assessing global BPF biodegradation potential. Soils represent a major sink for BPs, and BPF is frequently detected in these systems [64, 65]. This context leads to the hypothesis that soil microbial communities harbor diverse functional taxa capable of BPF degradation. Given that SMAG represents the most comprehensive soil-specific metagenomic resource to date [32], the BpfA reference sequence was mapped against species-level genomes in GTDB and SGBs to characterize the distribution of BpfA homologs across diverse microbial lineages. Among genomes representing 153 143 microbial species (spanning 12 phyla), 1652 genomes (1.1%) contained BpfA homologs. In total, 67.4% of the microbial species potentially containing the *bpfA* remained uncultured (Fig. S9). BpfA homologs were frequently found among *Pseudomonadota* (70.0%) and *Actinomycetota* (20.5%), with no archaeal representatives detected (Fig. 6A). At the genus level, *Paraburkholderia*, *Novosphingobium*, and *Sphingobium* constituted the major *bpfA*-harboring taxa, whereas only a few strains within the genus *Microbacterium* harbor this gene (Fig. 6B). Combining previously reported BPF-degrading strains, it can be inferred that bacteria carrying *bpfA* and involved in BPF degradation were widely distributed across *Pseudomonadota* and *Actinomycetota*. These results expand our knowledge of the variety of bacteria that participate in BPF biodegradation. Understanding the diversity and distribution of these bacteria is essential for elucidating their ecological importance and potential applications.

The environmental distribution of *bpfA* was determined by mapping reference sequences to 40 039 MAGs derived from 3304 global metagenomes. Based on habitat metadata, MAGs harboring *bpfA* were classified into eight ecosystem types: cultivated land, forest, artificial surfaces, wetland, grassland, bare land, shrubland, and tundra (Fig. 6C). Cultivated land and forest contained the highest numbers of positive MAGs (179 and 78, respectively), with relative abundances of 1.5% and 1.7% among total MAGs per ecosystem, respectively (Table S7). Globally, cultivated land in coastal regions of China showed the highest abundance of BpfA homologs (Fig. 6C). This is primarily due to the high population density and economic development in coastal cities, which drive a significant demand for plastics and a concentration of industries such as epoxy resin and thermal paper enterprises [64]. Unintentional release of BPs during plastic production and use leads to environmental contamination, with subsequent migration and accumulation in soils. In addition, the application of sludge containing BPs also exacerbates soil pollution [65]. Consistent with this, a total of 32 types of BPs have been widely detected in the surface soils of China at total concentrations ranging from 0.387 to 713 ng·g^−1^, showing a stepwise decrease from the southeastern coast to inland regions [64]. Moreover, a total of 12 types of BPs were detected in 29 agricultural and urban soil samples from 21 provinces in China, with BPF reaching a high content of 212.9 ng·g^−1^ dry weight [65]. Long-term BPF exposure in soils may select for microbial communities enriched in degradation genes. A critical data gap exists between pollutant quantification and genomic profiling: quantitative BPF contamination data are absent for most metagenomes, while existing BPF monitoring records lack corresponding metagenomic information. This disjunction currently precludes robust correlation analyses between *bpfA* abundance and environmental BPF concentrations. Additionally, the higher abundance of BpfA homologs in US and Swiss forests (Fig. 6C) may be due to the abundance of lignin in these habitats, with the abundance of lignin degradation-related genes such as *eugO* and *vaO* also increasing accordingly [66].

## Conclusions

Given the widespread use of BPF, the residues of BPF in the environment cannot be overlooked; however, its microbial metabolic mechanism is currently poorly understood. In this study, the flavoprotein oxidase gene *bpfA* from *Microbacterium* sp. F2 was cloned and identified, and found to be responsible for catalyzing BPF into DHBP through a three-step reaction. BpfA exhibited the highest catalytic efficiency against BPF (*k*_cat_/*K*_m_ = 508.1 mM^−1^·s^−1^) among the substrates tested, with lower efficiencies against other 4-alkylphenol compounds such as VA (3.3 mM^−1^·s^−1^), 4PG (224.4 mM^−1^·s^−1^), and 4MOP (4.0 mM^−1^·s^−1^). In addition, BpfA homologs were found in 1.1% of microbial genomes spanning 12 phyla within the SMAG database. This study deepens our understanding of the BPF microbial degradation mechanism, revealing the catalytic function of the oxidase gene *bpfA* and its distribution in the environment. The high catalytic efficiency of BpfA and its activity against multiple substrates suggest its potential utility as a biocatalyst in bioremediation and synthetic biology applications.

## Supplementary Material

Supplemental_Materialal_wraf206

## Data Availability

The sequences of BpfA and FBO have been deposited into the GenBank database under accession numbers PV658135 and PV658134, respectively. The genome assembly data of *Microbacterium* sp. F2 have been deposited into the GenBank database under accession number JBNVBV000000000. All other data generated or analysed during this study are included in this published article and its supplementary material files.
