# Supplementary material for: Widespread distribution of BpfA-mediated bisphenol F degradation among members of the Pseudomonadota and Actinomycetota
Source: ISME J. 2025 Sep 15;19(1):wraf206. doi: 10.1093/ismejo/wraf206 (PMC12507022; doi:10.1093/ismejo/wraf206)
Supplement: Supplemental_Materialal_wraf206 [file supplemental_materialal_wraf206.docx]

Supplemental Material

Widespread distribution of BpfA-mediated bisphenol F degradation among members of the *Pseudomonadota* and *Actinomycetota*

Running title：The bisphenol F oxidase BpfA

Mingliang Zhang^a,1^, Changchang Wang^a,1^, Yanni Huang^a^, Qian Li^a^, Junqiang Hu^a^, Kaihua Pan^a^, Qian Zhu^a^, Wankui Jiang^b^, Jiguo Qiu^a^, Xin Yan^a*^, Qing Hong^a*^

^a^ Department of Microbiology, College of Life Sciences, Nanjing Agricultural University, Key Laboratory of Agricultural and Environmental Microbiology, Ministry of Agriculture and Rural Affairs, Nanjing 210095, China.

^b^ State Key Laboratory of Materials-Oriented Chemical Engineering, College of Biotechnology and Pharmaceutical Engineering, Nanjing Tech University, Nanjing 211800, PR China.

^1^ Contributed equally to this paper.

* Author for correspondence: Xin Yan and Qing Hong

E-mail address: yanxin@njau.edu.cn. and [hongqing@njau.edu.cn](mailto:hongqing@njau.edu.cn).

Tel: +86-25-84396685, Fax: +86-25-84395326

Physical mailing address: Qing Hong, College of Life Sciences, Nanjing Agricultural University, 1 Weigang, Xuanwu District, Nanjing, Jiangsu Province, 210095, China.

**
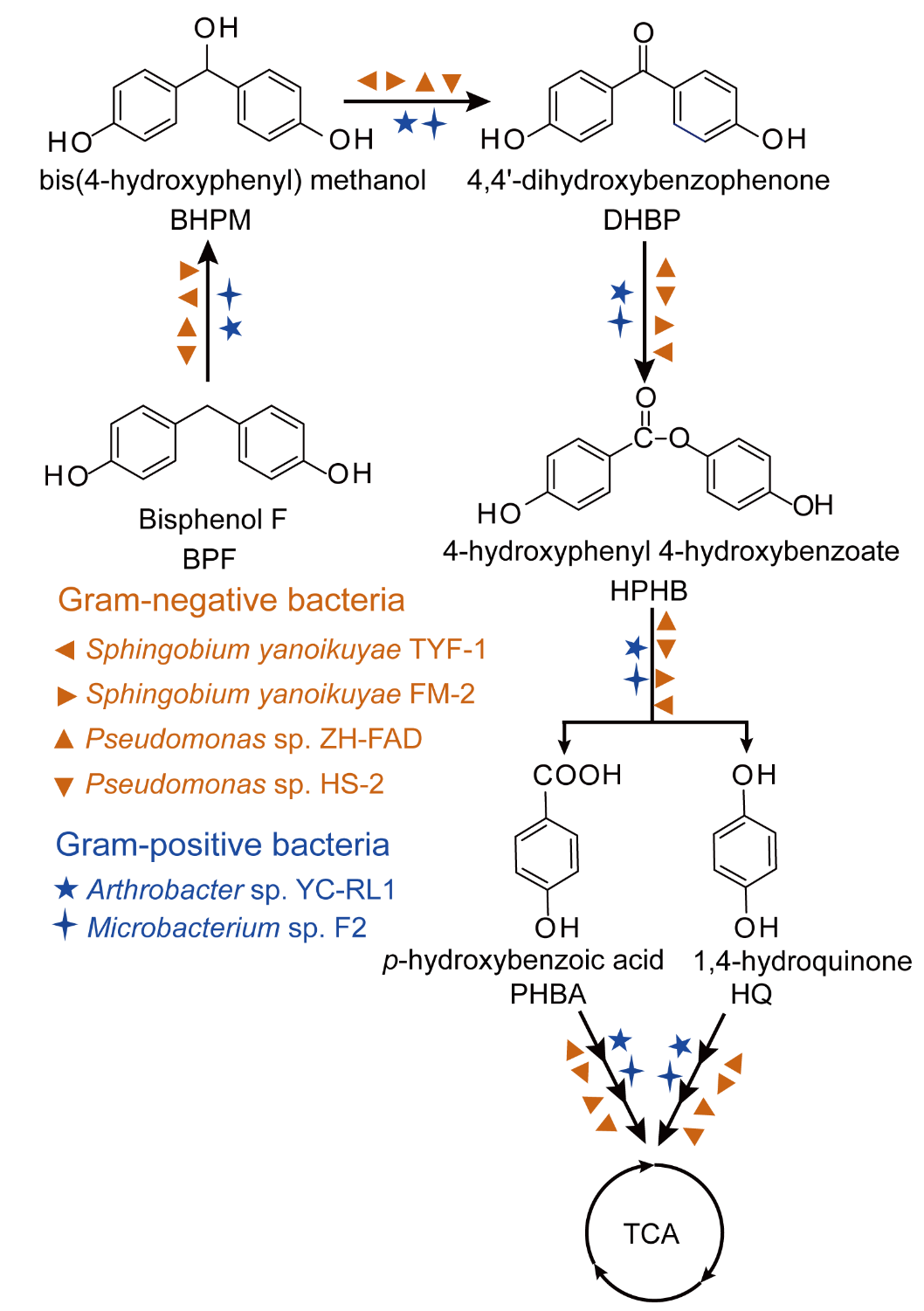
**

Fig S1. The conserved BPF degradation pathway in the reported strains.

*Pseudomonas* sp. HS-2 [16], *Pseudomonas* sp. ZH-FAD [17], *Sphingobium* *yanoikuyae* FM-2 [18], *Sphingobium yanoikuyae* TYF-1 [19], *Arthrobacter* sp. YC-RL1 [20], and *Microbacterium* sp. F2 [24].


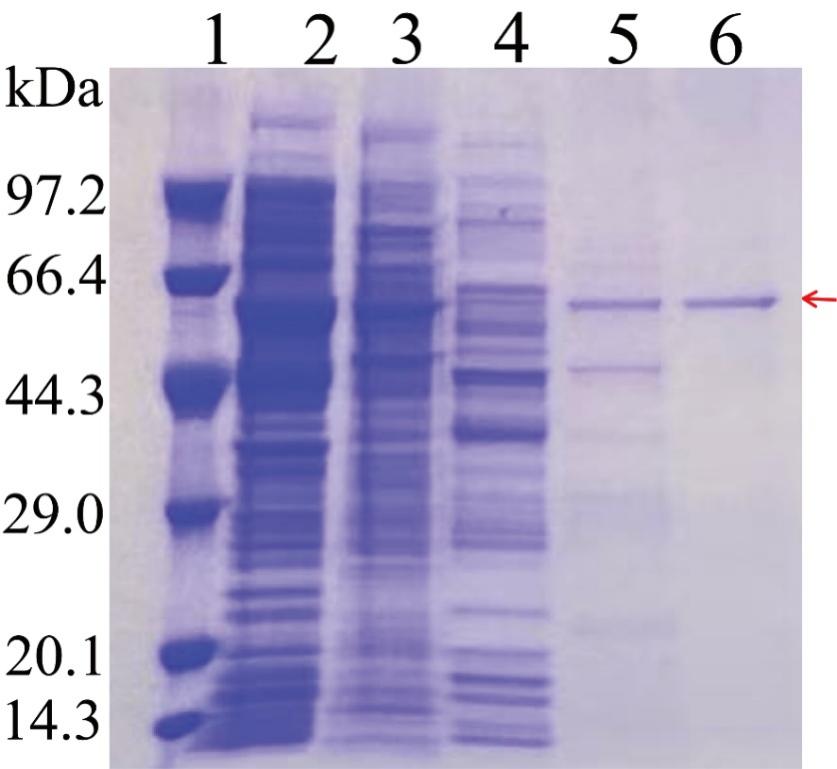


Fig S2. The purification of BpfA from strain F2 on SDS-PAGE. Lane 1: protein marker, Lane 2: cell extract, Lane 3: after ammonium sulfate precipitation, Lane 4: after by DEAE-Sepharose fast flow anion exchange chromatography, Lane 5: after Q-Sepharose fast flow anion exchange chromatography, Lane 6: after concentration by Microcon centrifugal filters and Sephadex-200 gel chromatography. The protein band (red arrow) were then excised and analyzed by matrix-assisted laser desorption ionization-time-of-flight (MALDI-TOF) mass spectrometry. Cell extract and ammonium sulfate precipitation fraction were diluted 10 and 4 times, respectively.


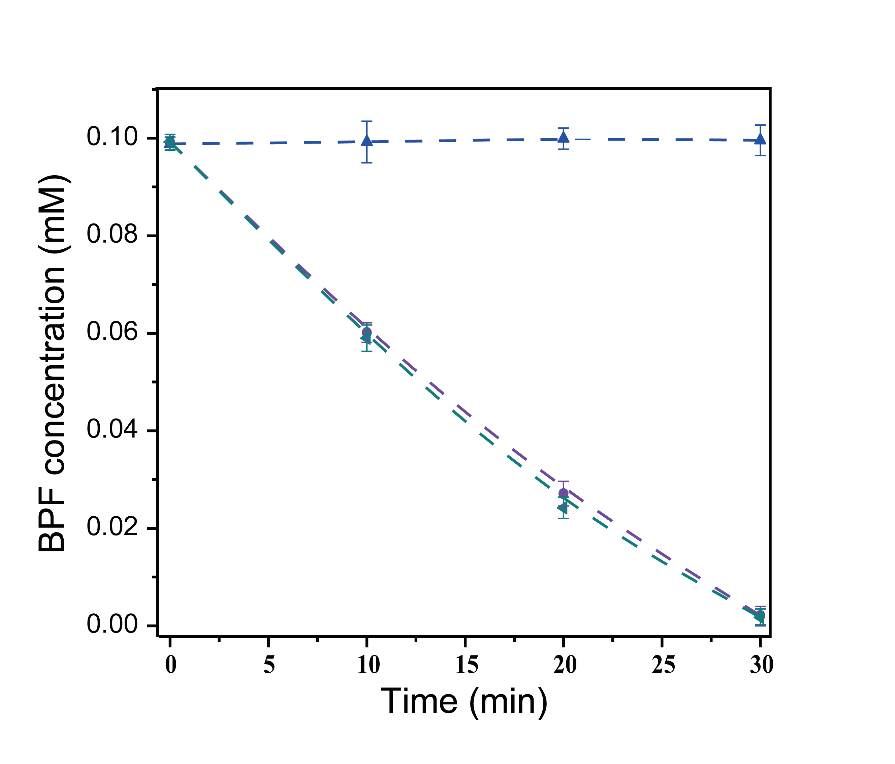


Fig S3. BPF degradation by the cell extracts of BPF-induced strain F2.
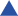
: Control;
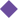
: cell extract of BPF-induced strain F2;
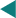
: cell extract of BPF-induced strain F2 with FAD, NADPH, and NADH. Error bars represent the standard error of three replicates.


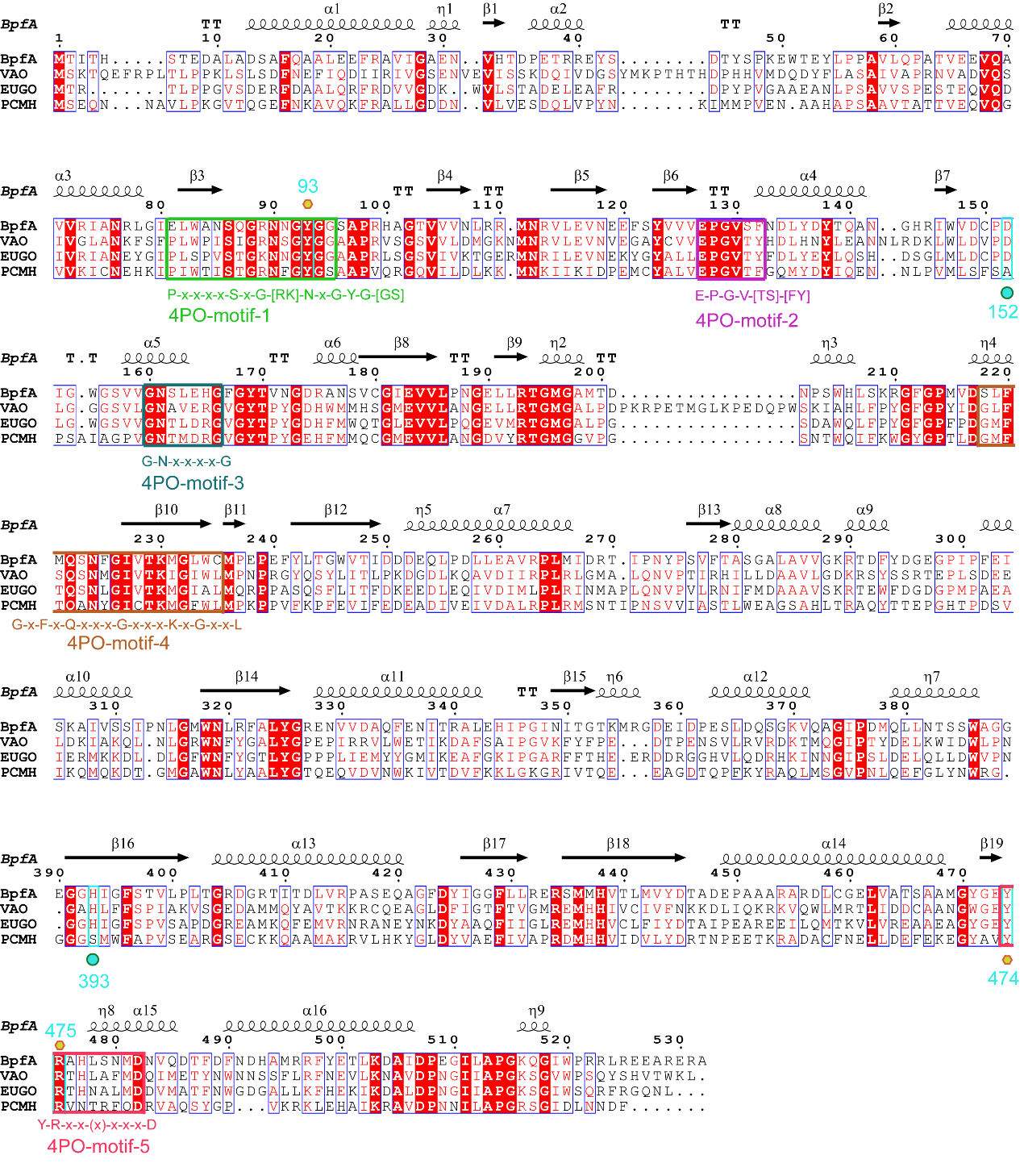


Fig S4. Sequence alignment of BpfA with the other members of the 4PO subgroup (EUGO, PCMH, and VAO). The five motifs of 4PO are labeled in different colors below the sequence. Pentagon represent the position of catalytic Tyr-Tyr-Arg (Tyr93, Tyr474, and Arg475) residues in BpfA. The FAD-binding site (Asp152 and His393) are denoted by circle.


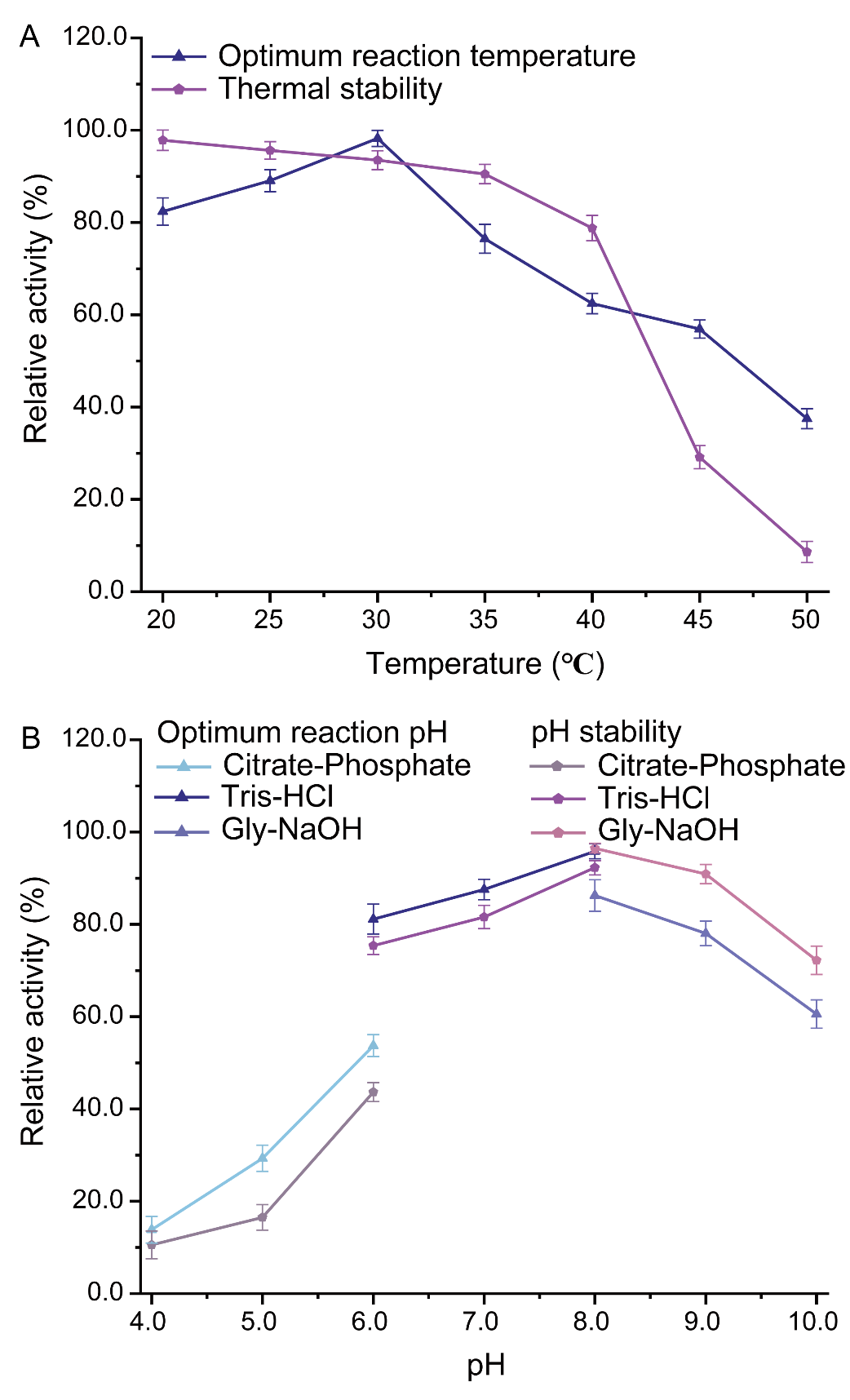


Fig S5. Effects of temperature and pH on the enzyme activity and stability of BpfA. The optimal temperature and thermal stability for the activities of BpfA (A); The optimal pH and pH stability for the activities of BpfA (B). The optimal temperature of BpfA (A) were determined using 20 mM Tris-HCl buffer (pH 7.0) at 20-50 °C. The optimal pH of BpfA (B) was determined using 20 mM disodium hydrogen phosphate-citric acid buffer (pH 4.0 to 6.0), 20 mM Tris-HCl (pH 6.0 to 8.0) and 20 mM glycine-NaOH buffer (pH 8.0 to 10.0). The reaction without enzyme was used as a blank control. Error bars represent the standard error of three replicates.

**
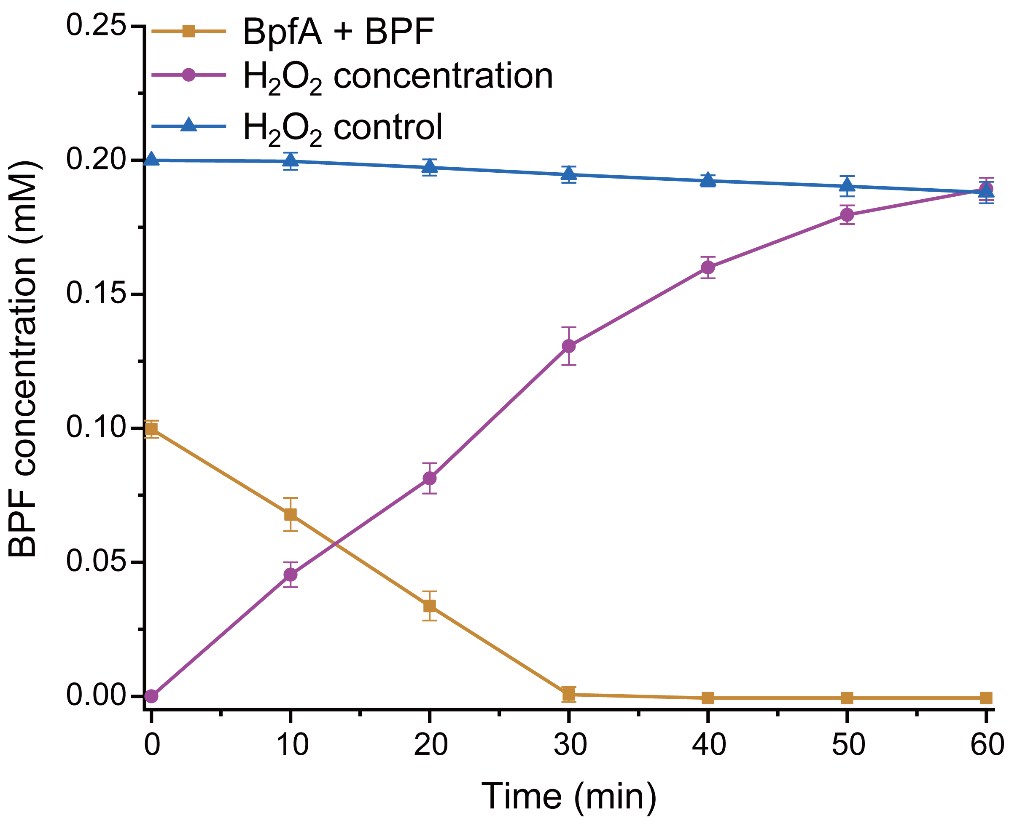
**

Fig S6. Hydrogen peroxide generated during the degradation of BPF. Orange represents the degradation of BPF by BpfA, and purple represents the generation of H_2_O_2_ during BPF degradation by BpfA. Bule represents the spontaneous degradation under the same conditions as 0.2 mM H_2_O_2_. Error bars represent the standard error of three replicates.


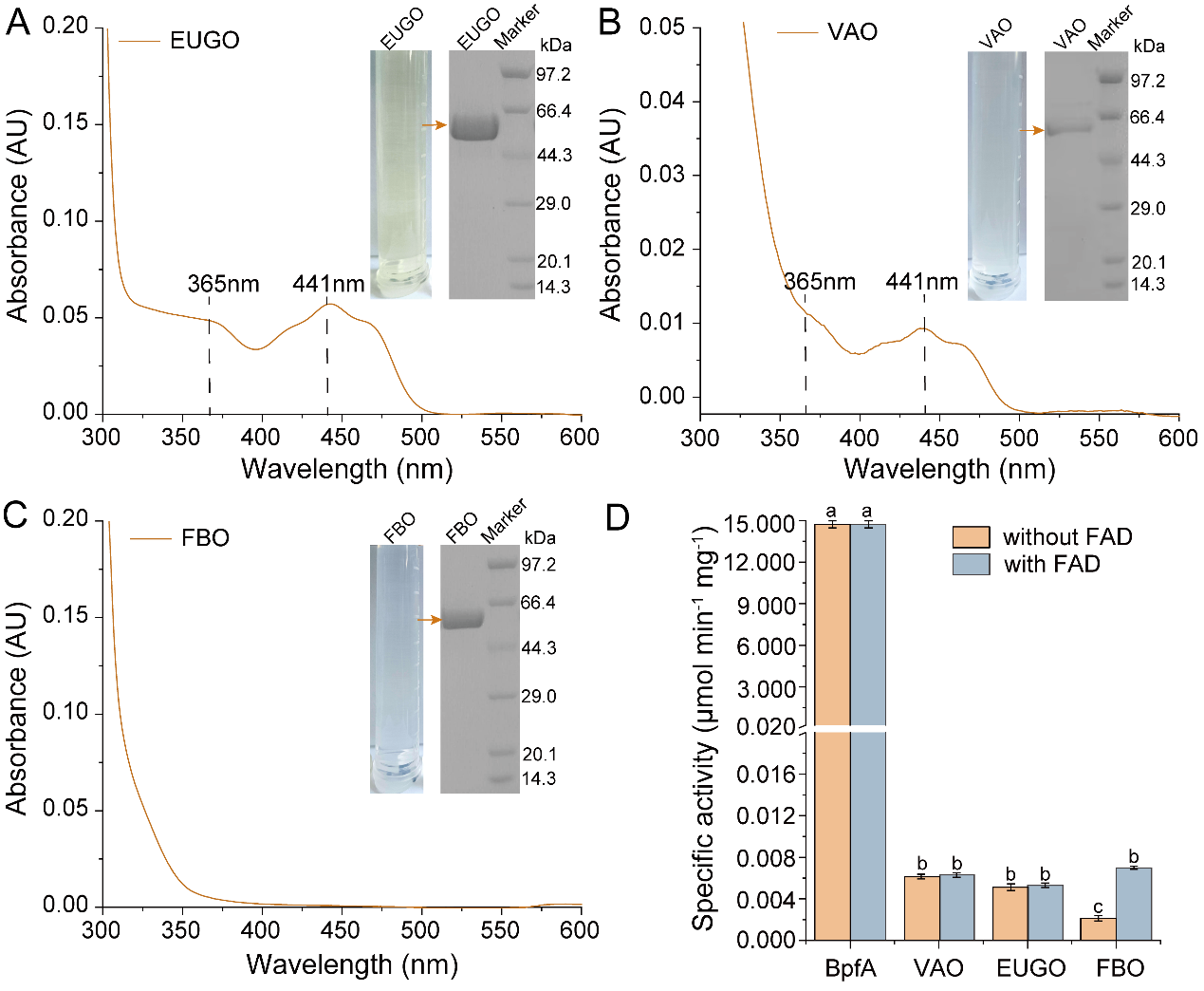


Fig S7. (A) UV-vis spectrum of native EUGO (0.65 mM). The inset shows SDS-PAGE analysis of the purified EUGO. (B) UV-vis spectrum of native VAO (0.02 mM). The inset shows SDS-PAGE analysis of the purified VAO. (C) UV-vis spectrum of native FBO (0.41 mM). The inset shows SDS-PAGE analysis of the purified FBO. (D) The specific activity of BpfA, VAO, EUGO, and FBO against BPF with or without FAD. These data represent the mean values of two replicates using independent enzyme preparation. Values with different lowercase letters are significantly different at *P* < 0.05 according to the LSD test.


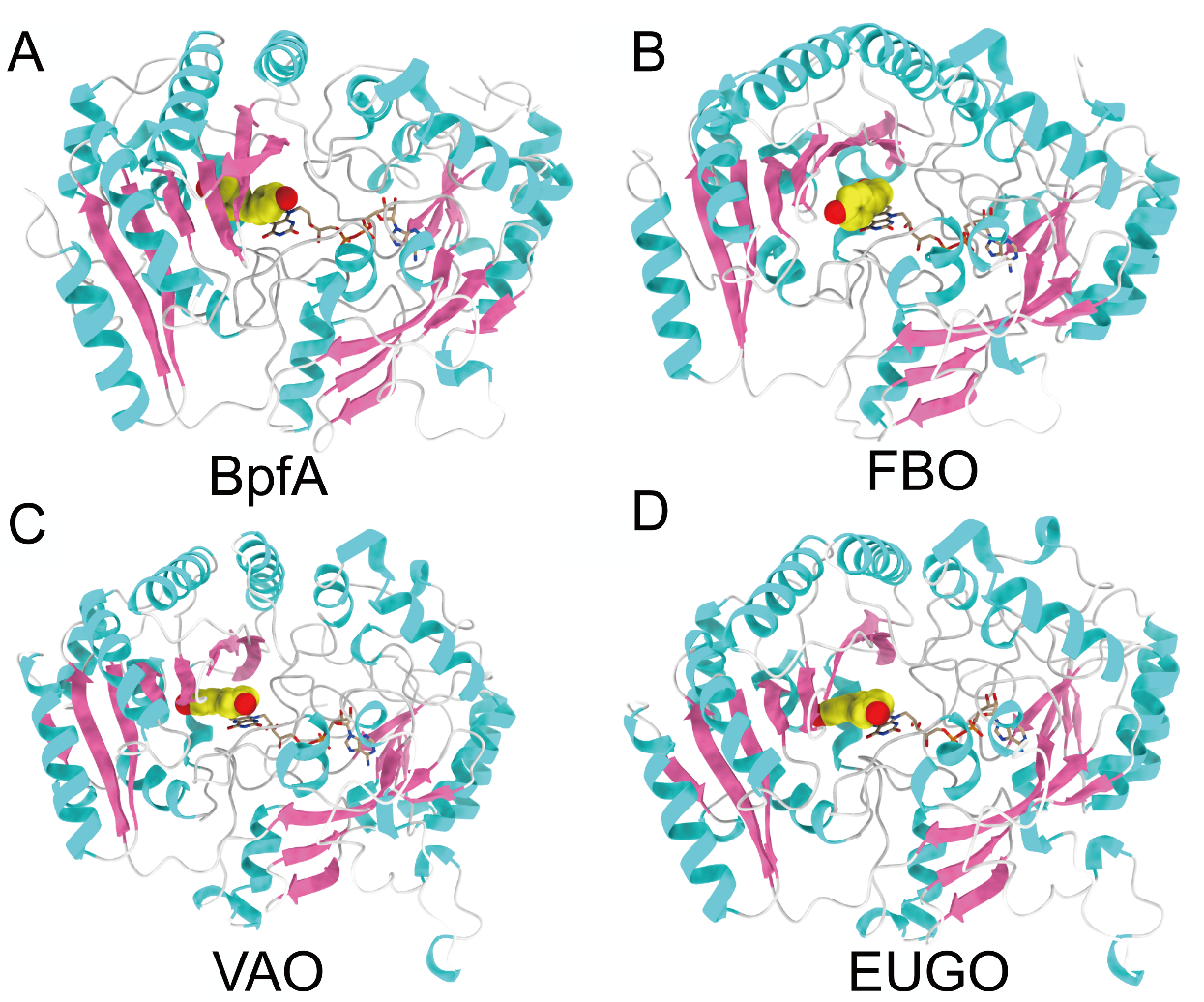


Fig S8. Molecular docking of BpfA (A), FBO (B), VAO (C), and EUGO (D) with BPF. The yellow spherical structure represents the substrate BPF, while the gray stick structure represents FAD. The names of the proteins are located below each structure.


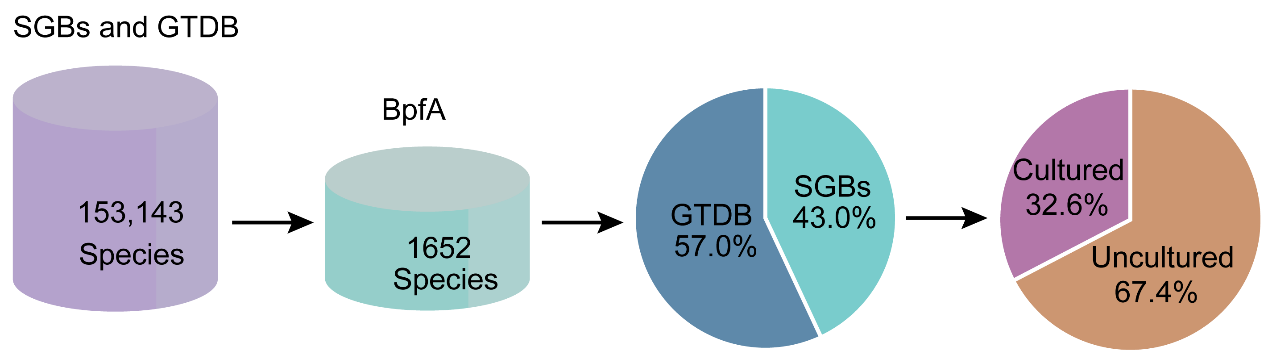


Fig S9. Species profiles with the BpfA from species-level genome bins (SGBs) and the Genome Taxonomy Database (GTDB).

Table S1 Physical and chemical properties of bisphenols.

| Compound (Abbreviation) | Chemical name | Molecular formula | Molecular weight | Boiling point | Melting point | pKa | log Kow | Structure |
| --- | --- | --- | --- | --- | --- | --- | --- | --- |
| BPA | 2,2-bis(4hydroxyphenyl)propane | C_15_H_16_O_2_ | 228.29 | 220 ℃ | 150 - 157 ℃ | 10.1 | 3.43 | 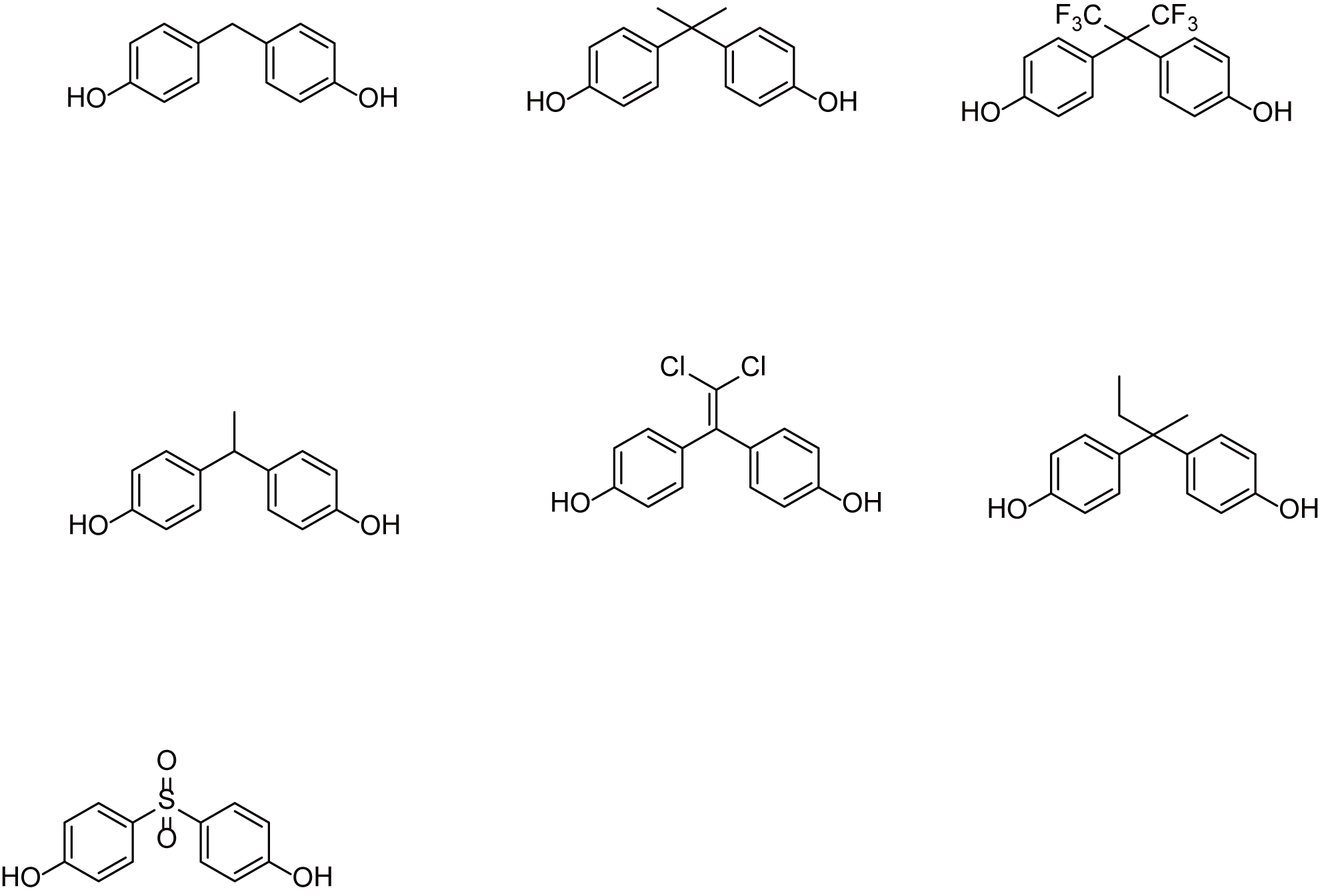 |
| BPB | 2,2-bis(4-hydroxyphenyl)butane | C_16_H_18_O | 242.31 | 412 - 414 ℃ | 138 - 140 ℃ | 10.1 | 4.13 | 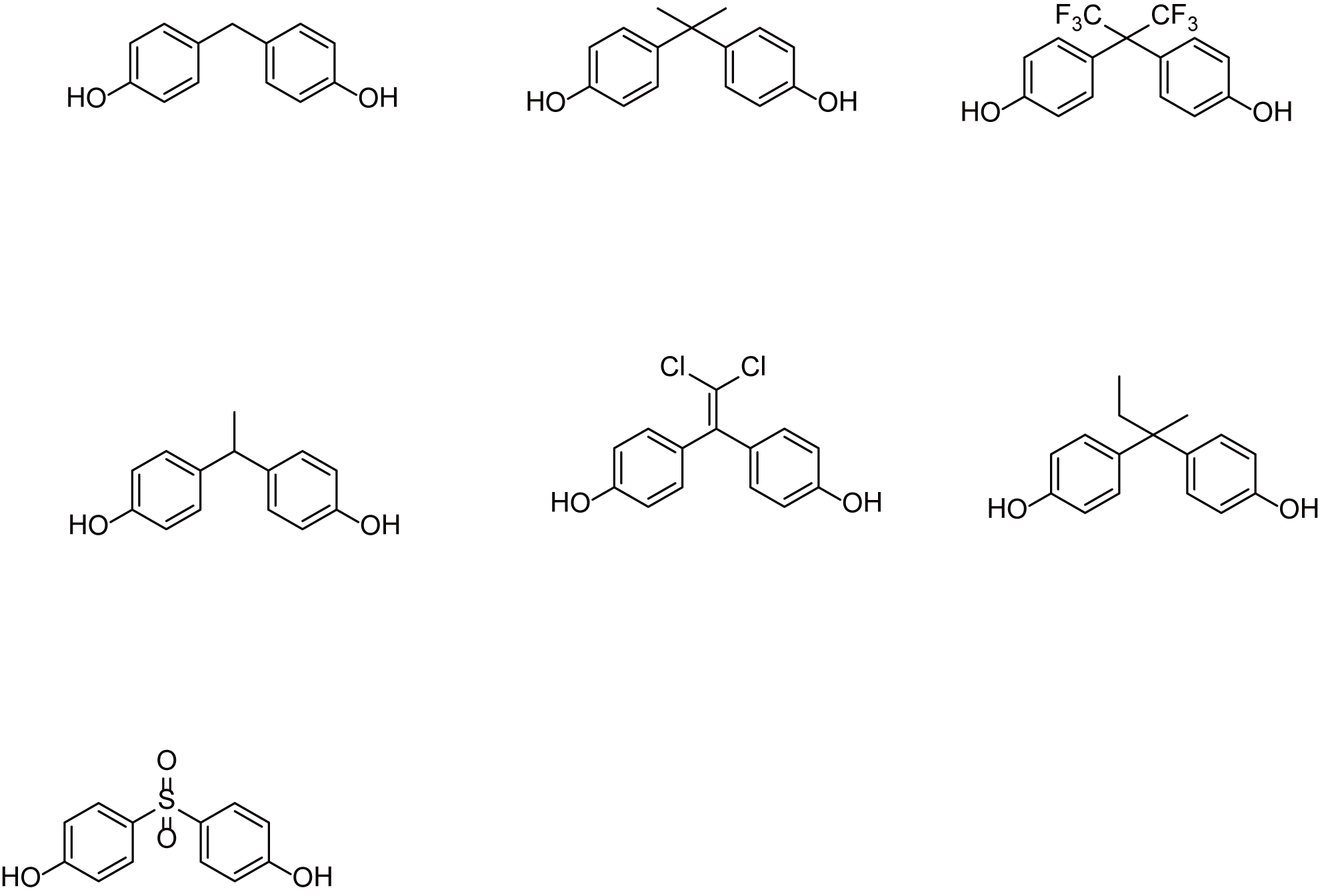 |
| BPC | 4,4’-(2,2-dichloroethene-1,1-diyl) diphenol | C_14_H_10_C_l2_O_2_ | 281.1 | 405 ℃ | 213 - 217 ℃ | 9.86 | - | 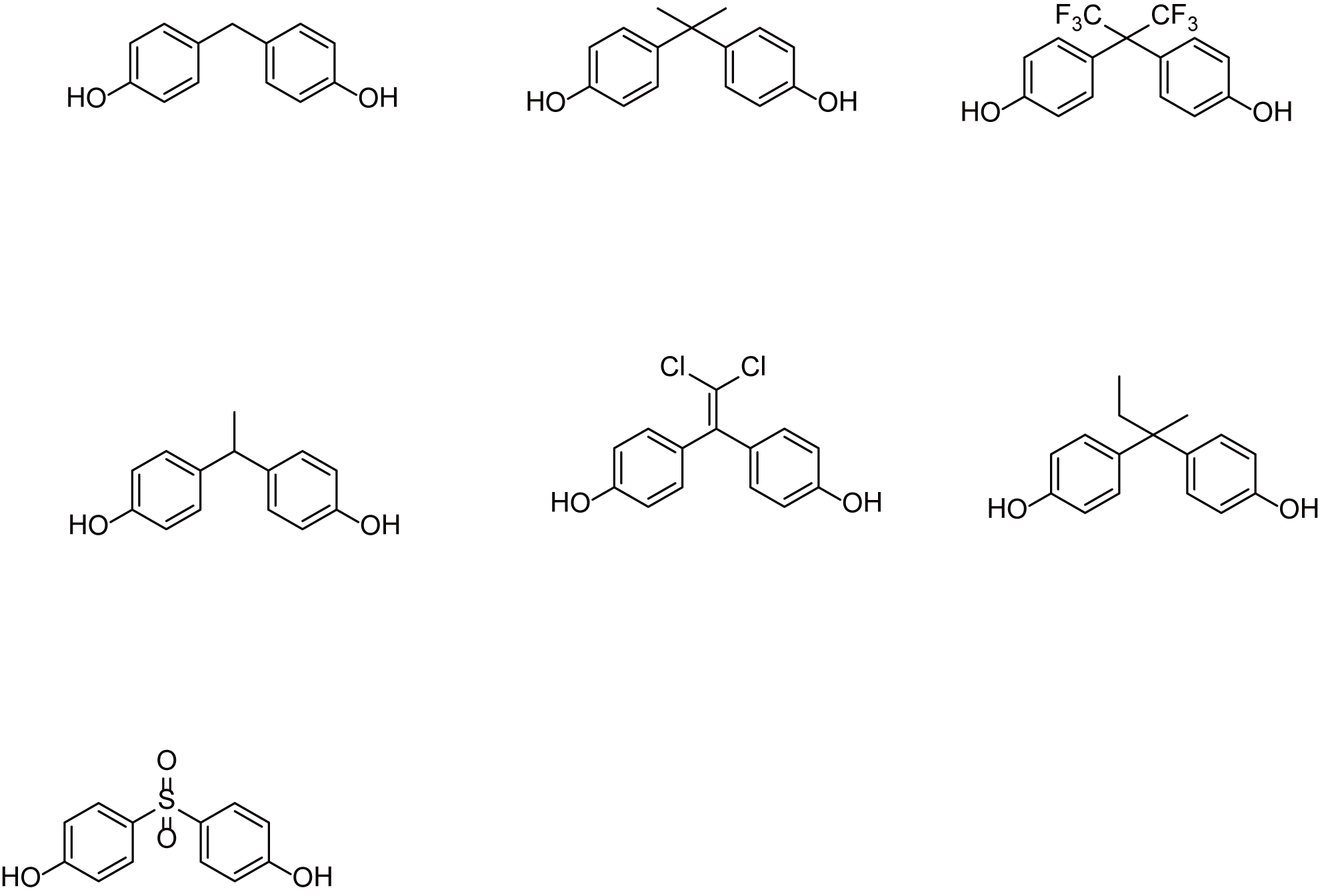 |
| BPE | 4,40-Ethylidenebisphenol | C_14_H_14_O_2_ | 214.26 | - | - | - | - | 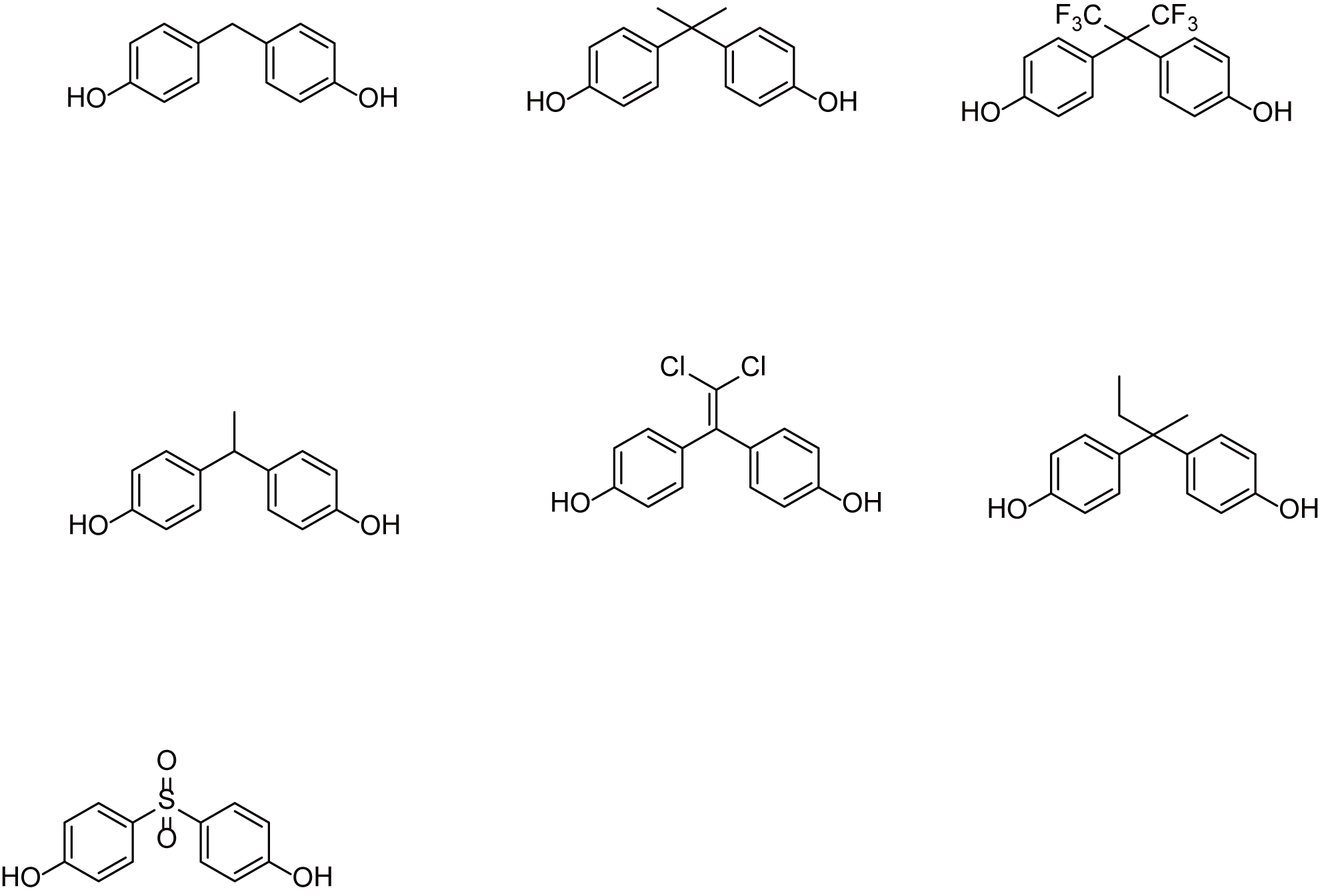 |
| BPF | 4,40-dihydroxydiphenylmethane | C_13_H_12_O_2_ | 200.23 | 389 - 390 ℃ | 128 - 130 ℃ | pKa_1_ = 7.55  pKa_2_ = 10.80 | 2.91 | 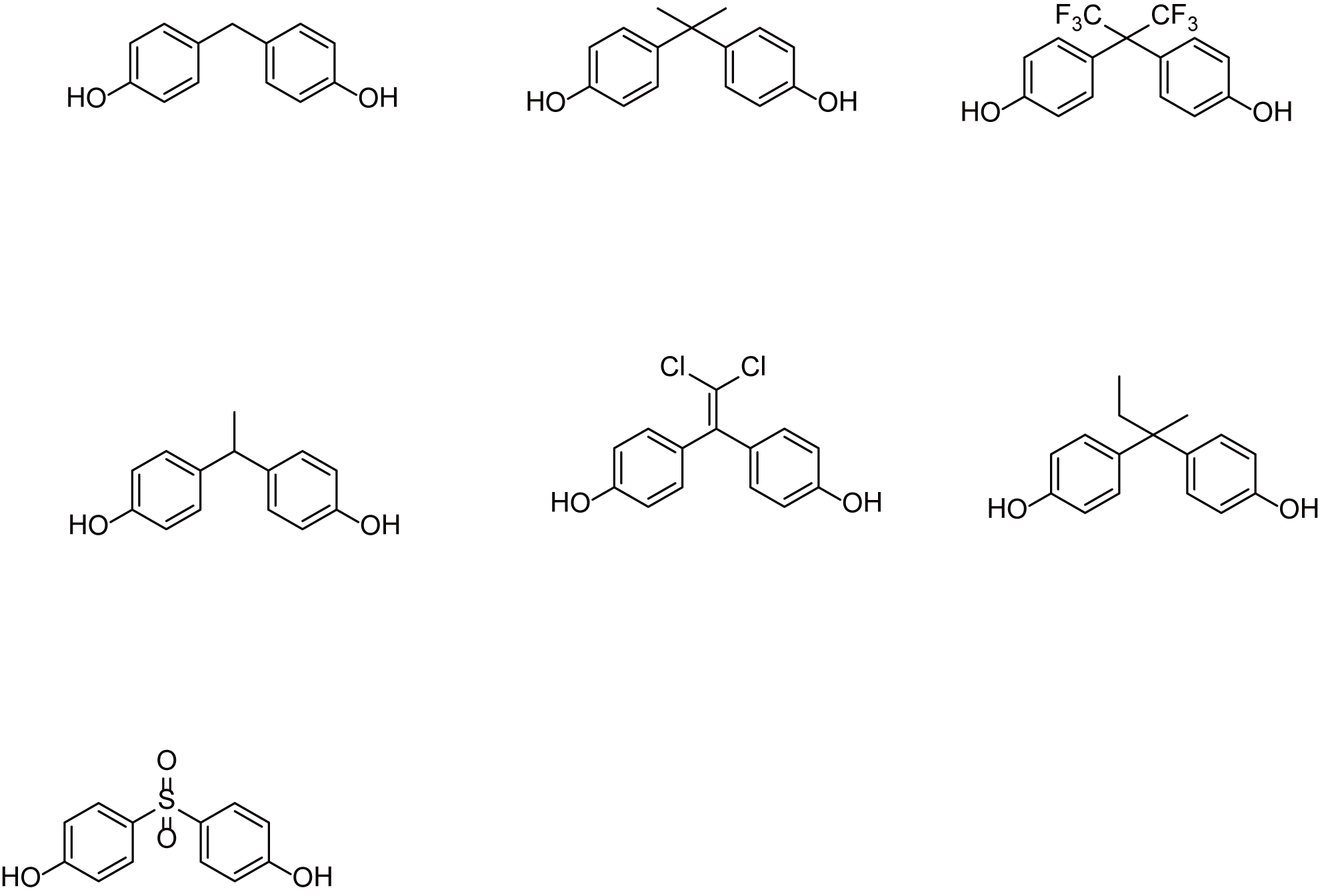 |
| BPS | 4,40-sulfonyldiphenol | C_12_H_10_O_4_S | 250.27 | 240 - 241 ℃ | 240.5 ℃ | 8.2 | 1.65 | 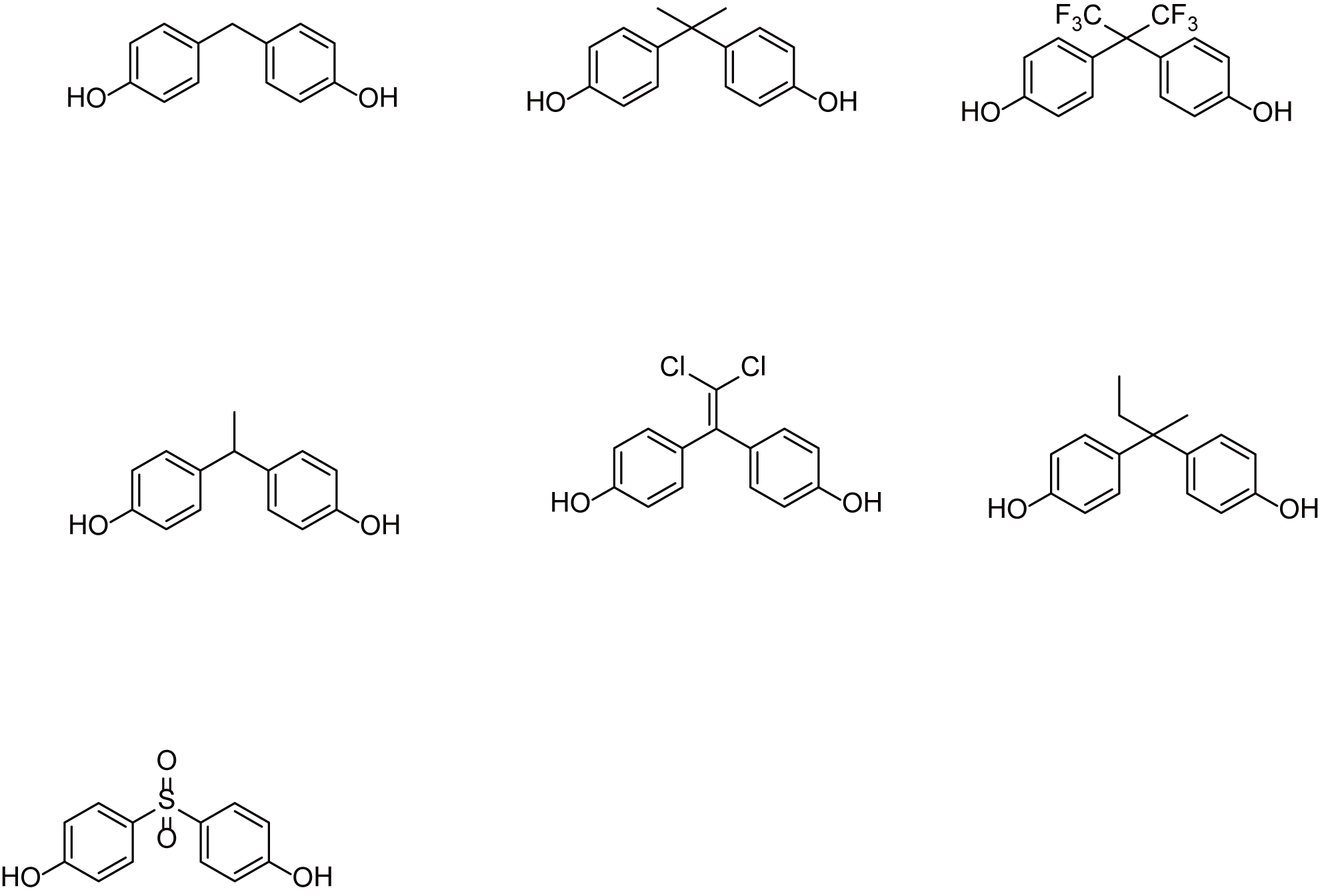 |
| BPAF | 4,4’-(hexafluoro-isopropylidene) diphenol | C_15_H_10_F_6_O | 336.23 | 400 ℃ | 125 - 126 ℃ | 9.2 | 4.47 | 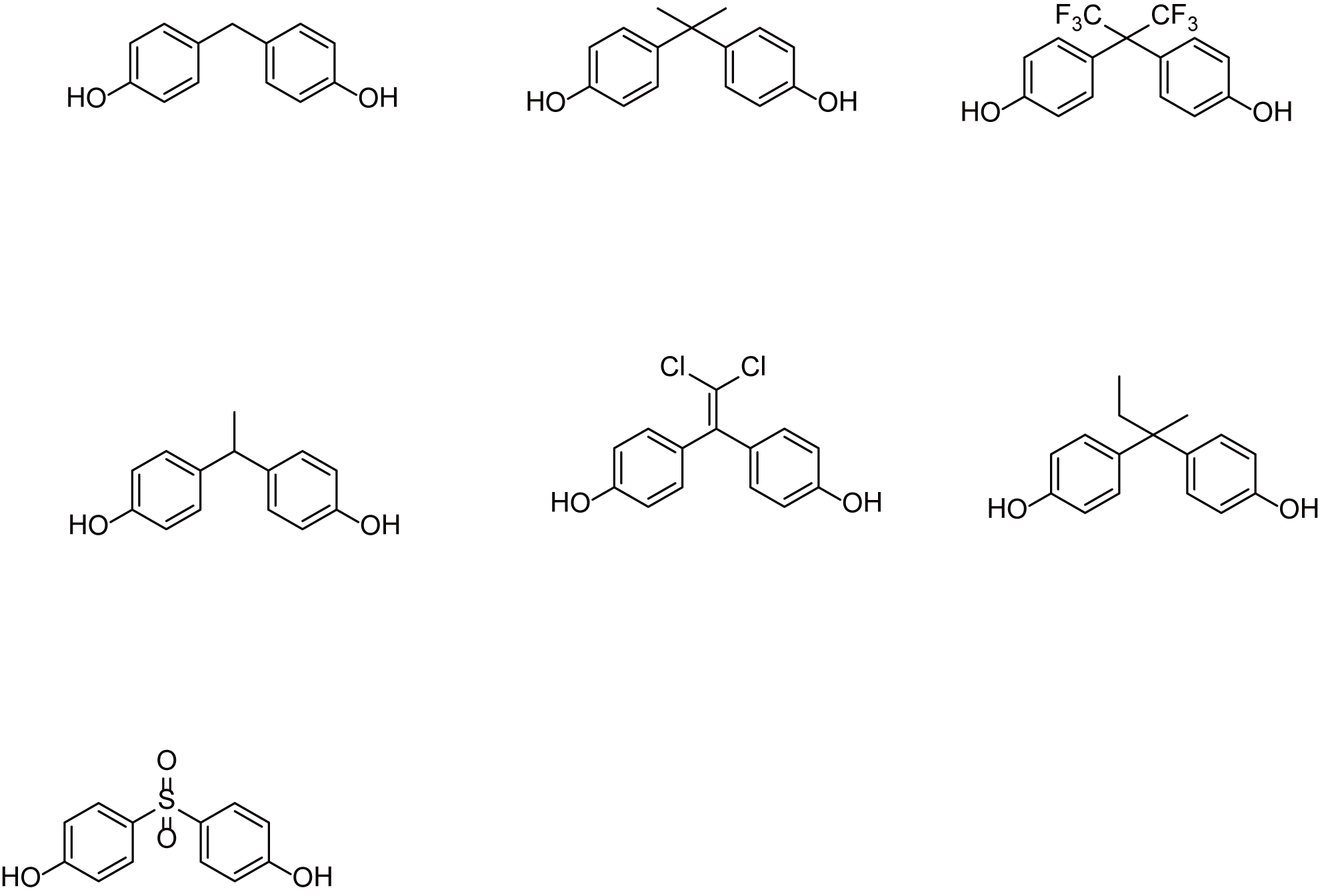 |

| Strains or plasmids | Description | Source |
| --- | --- | --- |
| Strains |  |  |
| *Microbacterium* sp. F2 | Degrades bisphenol F; Gram-positive, Wild type | This study |
| *E. coli* strains |  |  |
| BL21(DE3) | F^-^ *ompT hsdS_B_ (r_B_^-^ m_B_^-^) dcm gal* λ(DE3) | Vazyme |
| Plasmids |  |  |
| pET-29a(+) | Km^r^; Expression vector | Lab stock |
| pET*-bpfA*_F2_ | Km^r^; *NdeI-XhoI* fragment containing *bpfA*_F2_ gene inserted into pET29a(+) | This study |
| pET*-bpfA*_F2_^Y93A^ | Km^r^; *NdeI-XhoI* fragment containing *bpfA*_F2_^Y93A^ gene inserted into pET29a(+) | This study |
| pET*-bpfA*_F2_^Y474A^ | Km^r^; *NdeI-XhoI* fragment containing *bpfA*_F2_^Y474A^ gene inserted into pET29a(+) | This study |
| pET*-bpfA*_F2_^R475A^ | Km^r^; *NdeI-XhoI* fragment containing *bpfA*_F2_^R475A^ gene inserted into pET29a(+) | This study |
| pET*-bpfA*_F2_^D152A^ | Km^r^; *NdeI-XhoI* fragment containing *bpfA*_F2_^D152A^ gene inserted into pET29a(+) | This study |
| pET*-bpfA*_F2_^H393A^ | Km^r^; *NdeI-XhoI* fragment containing *bpfA*_F2_^H393A^ gene inserted into pET29a(+) | This study |
| pET-*fbO*_W15_ | Km^r^; *NdeI-XhoI* fragment containing *fbO*_W15_ gene inserted into pET29a(+) | This study |
| pET-*vaO* _CBS 170.90_ | Km^r^; *NdeI-XhoI* fragment containing *vaO* _CBS 170.90_ gene inserted into pET29a(+) | This study |
| pET-*eugO* _RHA1_ | Km^r^; *NdeI-XhoI* fragment containing *eugO* _RHA1_ gene inserted into pET29a(+) | This study |

Table S2 Strains and plasmids used in this study.

| Table S3 Primers used in this study. | | |
| --- | --- | --- |
| Primers | Sequence (5'–3') ^a^ | Description |
| *bpfA*_F2_-F | TAAGAAGGAGATATACATATGATGACCATCACCCATTCGACCGAGGACGC | To construct plasmid pET-*bpfA*_F2_ |
| *bpfA*_F2_-R | GTGGTGGTGGTGGTGCTCGAGTGCCCGCTCCCGCGCCTC |  |
| Y93A-F | GCAGGGCCGCAACAACGGCGCCGGCGGGTCCGCCCCGCG | To construct plasmid pET*-bpfA*_F2_^Y93A^ |
| Y93A-R | GCGCGGGGCGGACCCGCCGGCGCCGTTGTTGCGGCCCTGCGAGTTGGC |  |
| Y474A-F | GGCGATGGGCTACGGGGAGGCTCGCGCGCACCTGTCGAA | To construct plasmid pET*-bpfA*_F2_^Y474A^ |
| Y474A-R | GTTCGACAGGTGCGCGCGAGCCTCCCCGTAGCCCATCGC |  |
| R475A-F | GATGGGCTACGGGGAGTATGCCGCGCACCTGTCGAACAT | To construct plasmid pET*-bpfA*_F2_^R475A^ |
| R475A-R | CATGTTCGACAGGTGCGCGGCATACTCCCCGTAGCCCATCG |  |
| D152A-F | CATCTGGGTCGACTGCCCCGCCATCGGGTGGGGGAGCGT | To construct plasmid pET*-bpfA*_F2_^D152A^ |
| D152A-R | CGACGCTCCCCCACCCGATGGCGGGGCAGTCGACCCAGATG |  |
| H393A-F | GGCGGGCGGGGAGGGCGGCGCCATCGGCTTCTCGACCGTGCTGCCGC | To construct plasmid pET*-bpfA*_F2_^H393A^ |
| H393A-R | CACGGTCGAGAAGCCGATGGCGCCGCCCTCCCCGCCCGC |  |
| *nofbO*_W15_-F | TAAGAAGGAGATATACATATGATGATGTCCGAACACTTGCCC | To construct plasmid pET-*fbO*_W15_ |
| *fbO*_W15_-R | GTGGTGGTGGTGGTGCTCGAGCAGATCGCCTCCCTTCCC |  |
| *eugO*_RHA1_-F | TCCGTCGACAAGCTTGCGGCCGCACTCGAGATGACAAGGACTCTACCCCCAG | To construct plasmid pET-*eugO*_RHA1_ |
| *eugO*_RHA1_-R | ATCTCAGTGGTGGTGGTGGTGGTGCTCGAGTTAGAGGTTCTGACCGCGGAAAC |  |
| *vaO*_CBS 170.90_-F | GTGGTGGTGGTGGTGCTCGAGCAGTTTCCAGCCCACTTG | To construct plasmid pET-*vaO*_CBS 170.90_ |
| *vaO*_CBS 170.90_-R | TAAGAAGGAGATATACATATGATGTCCAAAGCCTCCCCG |  |

Table S4 Purification of BpfA from strain F2

| Step | Total protein (mg) | Total activity  (U) | Specific activity  (U/mg) | Recovery  (%) | Fold |
| --- | --- | --- | --- | --- | --- |
| Cell extract | 159.03 | 58.84 | 0.37 | 100.0 | 1.00 |
| Ammonium sulfate precipitation | 20.89 | 31.13 | 1.49 | 52.9 | 2.51 |
| DEAE-Sepharose chromatography | 8.51 | 20.59 | 2.42 | 35.0 | 6.34 |
| Q-Sepharose chromatography | 1.83 | 13.89 | 7.59 | 23.6 | 13.74 |
| Sephadex-200 gel chromatography | 0.19 | 2.80 | 14.74 | 4.8 | 24.15 |

Table S5 The result of peptide mass spectrometry analysis of protein band

| ORF no.  (locus_tag) | Location | Peptides^a^ | PSMs^b^ | AAs^c^ | MW [kDa] | Score^d^ | Homologous protein^e^ | GenBank  accession no. | Identity  (%) |
| --- | --- | --- | --- | --- | --- | --- | --- | --- | --- |
| 1733 (ACQVDU_06965) | 1404956-1406554 | 23 | 83 | 532 | 58.80 | 188.39 | flavoprotein oxidase [*Mycolicibacterium*] | WP_226519350 | 54.4% |
| 1280 (ACQVDU_15455) | 3130960-3132243 | 24 | 82 | 427 | 445.20 | 170.48 | NAD(P)/FAD-dependent oxidoreductase [*Microbacterium*] | WP_292728035 | 100.0% |
| 1054 (ACQVDU_14355) | 2941356-2942783 | 20 | 74 | 475 | 54.00 | 154.82 | Rieske 2Fe-2S domain-containing protein [*Micrococcales*] | WP_228164524 | 100.0% |
| 3004 (ACQVDU_13245) | 2725684-2726598 | 15 | 63 | 304 | 32.10 | 129.57 | DMT family transporter [*Microbacterium*] | WP_228178617 | 93.1% |
| 2349 (ACQVDU_09975) | 2025295-2026383 | 13 | 45 | 362 | 37.60 | 111.50 | threonine synthase [*Microbacterium*] | WP_300594141 | 100.0% |
| 2584 (ACQVDU_11170) | 2277460-2278902 | 7 | 32 | 480 | 52.90 | 89.93 | cytochrome ubiquinol oxidase subunit I [*Microbacterium*] | WP_300591869 | 99.8% |
| 803 (ACQVDU_04060) | 823821-824276 | 8 | 30 | 151 | 15.90 | 80.38 | type II 3-dehydroquinate dehydratase [*Microbacterium*] | WP_300592868 | 100.0% |
| 1724 (ACQVDU_06920) | 1393943-1395784 | 7 | 34 | 613 | 68.30 | 67.39 | PEP-utilizing enzyme [*Microbacterium*] | WP_336401120 | 88.9% |
| 1704 (ACQVDU_06830) | 1378368-1379459 | 9 | 27 | 363 | 37.9 | 54.91 | glycerol dehydrogenase [*Herbiconiux*] | WP_092551598 | 58.5% |
| 2521 (ACQVDU_10845) | 2213638-2215359 | 10 | 29 | 573 | 60.40 | 43.57 | acyl-CoA dehydrogenase [*Microbacterium*] | WP_331791808 | 98.6% |
| 1242 (ACQVDU_15265) | 3100608-3102155 | 5 | 34 | 515 | 54.80 | 37.58 | alpha/beta hydrolase [*Microbacterium*] | WP_331791728 | 99.6% |
| 2441 (ACQVDU_10450) | 2126855-2128228 | 10 | 22 | 457 | 50.4 | 29.95 | FAD-dependent oxidoreductase [*Microbacterium*] | WP_292710698 | 100.0% |
| 308 (ACQVDU_01550) | 315433-316728 | 7 | 24 | 431 | 48.00 | 26.10 | citrate synthase [*Microbacterium*] | WP_363497997 | 99.5% |
| 2404 (ACQVDU_10260) | 2089875-2090711 | 7 | 26 | 278 | 28.40 | 21.48 | pyrroline-5-carboxylate reductase [*Microbacterium*] | WP_300591385 | 98.6% |
| 2612 (ACQVDU_11315) | 2307237-2308601 | 8 | 20 | 454 | 46.8 | 15.93 | 3-phosphoshikimate 1-carboxyvinyltransferase [*Microbacterium*] | WP_363495956 | 97.4% |

a. Number of different peptides identified. The higher the number, the higher the protein abundance.

b. Number of peptides matched to secondary spectra.

c. Number of amino acids.

d. Protein matching score, the higher the score, the higher the confidence

e. The top BLASTP hit was selected from NCBI Non-redundant Protein Sequences Database.

Table S6 Kinetic constants of BpfA, VAO, EUGO, and FBO

| Protein | BPF | | | 4MOP | | | VA | | | 4PG | | |
| --- | --- | --- | --- | --- | --- | --- | --- | --- | --- | --- | --- | --- |
|  | *K*_m_ (μM) | *k_cat_* (s^-1^) | *k*_cat_/*K*_m_ (mM^-1^ s^-1^) | *K*_m_ (μM) | *k*_cat_ (s^-1^) | *k*_cat_/*K*_m_ (mM^-1^ s^-1^) | *K*_m_ (μM) | *k*_cat_ (s^-1^) | *k*_cat_/*K*_m_ (mM^-1^ s^-1^) | *K*_m_ (μM) | *k*_cat_ (s^-1^) | *k*_cat_/*K*_m_ (mM^-1^ s^-1^) |
| BpfA | 12.4 ± 1.9 | 6.3 ± 0.01 | 508.1 a | 297.4 ± 5.0 | 1.2 ± 0.002 | 4.0 b | 269.5 ± 10.3 | 0.9 ± 0.005 | 3.3 c | 25.4 ± 2.9 | 5.7 ± 0.04 | 224.4 a |
| VAO | 205.6 ± 18.5 | 0.05 ± 0.001 | 0.2 b | 72.0 ± 7.1 | 3.9 ± 0.009 | 54.2 a | 140 ± 12.3 | 6.0 ± 0.2 | 42.9 b | 71.4 ± 11.6 | 5.0 ± 0.02 | 70.4 b |
| EUGO | 185.1 ± 11.3 | 0.04 ± 0.003 | 0.2 b | 73.2 ± 1.5 | 4.1 ± 0.01 | 56.0 a | 89.6 ± 11.3 | 5.9 ± 0.3 | 65.8 a | 59.3 ± 0.7 | 4.8 ± 0.002 | 80.9 b |
| FBO | 167.3 ± 12.8 | 0.05 ± 0.0002 | 0.3 b | - | - | - | 182.6 ± 9.3 | 0.02 ± 0.0001 | 0.1 d | 170.0 ± 5.9 | 0.03 ± 0.0001 | 0.2 c |

-, means no detectable activity.

Table S7 The relative abundance of *bpfA* gene-containing MAGs across ecosystems

| Ecosystem | Gene number^a^ | MAGs number^b^ | Total MAGs number^c^ | Relative abundance^d^ |
| --- | --- | --- | --- | --- |
| Cultivated land | 369 | 179 | 12318 | 1.5% |
| Forest | 164 | 78 | 4560 | 1.7% |
| Artificial Surfaces | 250 | 68 | 6295 | 1.1% |
| Wetland | 206 | 42 | 4602 | 0.9% |
| Grassland | 101 | 41 | 6433 | 0.6% |
| Bare Land | 14 | 12 | 1620 | 0.7% |
| Shrubland | 17 | 8 | 808 | 1.0% |
| Tundra | 12 | 6 | 1874 | 0.3% |

a, The number of *bpfA* gene;

b, Number of MAGs harboring *bpfA* gene;

c, The total number of MAGs in the SMAG database;

d, Calculated as (MAGs number/Total MAGs number).
